# Machine learning with decision curve analysis evaluates nutritional metabolic biomarkers for cardiovascular-kidney-metabolic risk: an NHANES analysis

**DOI:** 10.3389/fnut.2025.1597864

**Published:** 2025-05-08

**Authors:** Jun Huang, Zhuo Liu, WeiPeng Feng, YuanLing Huang, XinChun Cheng

**Affiliations:** ^1^Graduate School, Xinjiang Medical University, Urumqi, China; ^2^People's Hospital of Xinjiang Uygur Autonomous Region, Urumqi, China; ^3^Shenzhen Institute of Information Technology, Shenzhen, China; ^4^Jiangsu Hengrui Pharmaceuticals Co., Ltd., Lianyungang, China

**Keywords:** Cardiovascular-Kidney-Metabolic Syndrome (CKM), machine learning, decision curve analysis (DCA), insulin resistance (IR), RAR, all-cause mortality

## Abstract

**Background:**

The American Heart Association recently introduced the concept of Cardiovascular-Kidney-Metabolic Syndrome (CKM), emphasizing the interplay between metabolic disorders, cardiovascular diseases, and kidney diseases. Although insulin resistance (IR) and chronic inflammation are core drivers of CKM, the relationships causing imbalance have not been fully evaluated. Emerging biomarkers (RAR, NPAR, SIRI, Homair) offer multidimensional prediction capabilities by simultaneously assessing nutritional metabolism, cellular inflammation, and insulin resistance in diabetes.

**Methods:**

This study included data from 19,884 participants in the National Health and Nutrition Examination Survey (NHANES) from 1999 to 2018. The study developed novel indices (RAR, NPAR, SIRI, Homair) and assessed their CKM predictive value through: Multivariable logistic/Cox regression; Restricted cubic splines; Machine learning (XGBoost, LightGBM); Decision curve analysis. Subgroup analyses were conducted to assess interactive effects on specific populations.

**Results:**

After weighted analysis, multi-model logistic regression showed that RAR, SIRI, NPAR, and Homair remained strongly correlated with CKM after adjusting for various factors (*p* < 0.05), with RAR showing the most pronounced relationship (OR: 2.73, 95% CI: 2.07–3.59, *p* < 0.001). RCS curves revealed nonlinear relationships between these factors and outcomes (nonlinear *p* < 0.05). In multi-model Cox regression, RAR, SIRI, and NPAR were associated with all-cause mortality (*p* < 0.05), and RAR was linked to all-cause, cardiovascular disease (CVD), and kidney disease mortality (*p* < 0.05), with the strongest link (OR: 2.38, 95% CI: 1.98–2.88, *p* < 0.001). Machine learning ranked RAR, SIRI, and Homair as top predictors for CKM diagnosis. The DCA model further validated these three Lasso-selected variables, showing clinical utility. The model combining RAR, diabetes mellitus (DM), and age demonstrated outstanding performance (AUC = 0.907), offering clinical reference value.

**Conclusion:**

This study demonstrates significant relationship between RAR, NPAR, SIRI, and Homair with the five stages of CKM, with RAR showing the robust association. DCA-confirmed RAR demonstrates high clinical translatability as a standalone predictor for CKM risk stratification.

## Introduction

CVD: CVD (1) is a leading cause of global morbidity and mortality, responsible for approximately 31% of all deaths worldwide. Risk factors include hypertension, dyslipidemia, smoking, and diabetes. The burden of CVD is expected to rise, particularly in low-and middle-income countries due to aging populations and increased prevalence of risk factors. CVD plays a key part to the global healthcare burden (2), with high costs related to treatment, hospitalizations, and long-term care.

Diabetes Mellitus (3) (DM): Diabetes mellitus, including both type 1 and type 2, is a chronic metabolic condition characterized by impaired insulin action or secretion. The global prevalence of diabetes has rapidly increased, with over 400 million people affected worldwide. Type 2 diabetes, driven by obesity and insulin resistance, accounts for approximately 90–95% of all diabetes cases. Diabetes increases the risk of cardiovascular disease, kidney failure, and neuropathy, contributing to a significant global health burden (4) and reduced quality of life.

Cardiovascular-Kidney-Metabolic Syndrome (5) (CKM): An emerging clinical syndrome characterized by the complex interaction of metabolic disorders, chronic inflammation, and multi-organ damage. Although the American Heart Association (AHA) clinical framework introduced CKM staging criteria in 2023, the current system mainly relies on traditional single-dimensional indicators: HbA1c for assessing metabolic control, and estimated glomerular filtration rate (eGFR) for reflecting kidney function. While these indicators are widely used, they have significant limitations—they cannot capture the dynamic imbalance among metabolism, inflammation, and nutrition, which is the core driving force behind CKM progression (6).

Against this backdrop, we have chosen the Red Cell Distribution Width-to-Albumin Ratio (RAR), Neutrophil-to-Total Protein Ratio (NPAR), Systemic Inflammation Response Index (SIRI), and Homair insulin resistance index as the focus of our study due to their unique multidimensional value.

The RAR (7) [RDW (%)/ALB (g/dL)] integrates oxidative stress (where increased RDW reflects disturbed red blood cell production and endothelial dysfunction) and the balance of nutrition and inflammation (low albumin levels suggest chronic inflammatory depletion). It can simultaneously assess vascular damage and protein-energy malnutrition, and has been shown to predict mortality in various diseases, including acute myocardial infarction, diabetes, and chronic kidney disease. This is more aligned with the chronic disease progression of CKM, compared to CRP, which primarily reflects acute inflammation.

The NPAR (8) [Neutrophil Percentage (%)/Albumin (g/dL)] combines innate immune activation with nutritional status, addressing the limitation of other indicators, such as NLR, that cannot assess the nutritional-metabolic imbalance. This index holds potential value in evaluating chronic diseases related to inflammation-nutrition imbalance, such as metabolic syndrome (9) and cardiovascular diseases (10).

The SIRI (11) is calculated from the counts of neutrophils, monocytes, and lymphocytes, and is used to quantify systemic inflammation levels. It dynamically monitors the imbalance between innate immunity (neutrophils/monocytes) and adaptive immunity (lymphocytes). Increased SIRI is closely associated with the risk and prognosis of inflammation-related diseases, such as infections, cancer, and cardiovascular diseases (12). It is commonly used as a marker for chronic low-grade inflammation and is more capable of identifying the low-grade sustained inflammation characteristic of CKM compared to traditional markers.

The Homair (13) index is based on fasting blood glucose and insulin levels, serving as a measure of insulin resistance. It is an important predictor of metabolic syndrome, type 2 diabetes, and obesity-related diseases. Compared to HbA1c, Homair provides a more accurate reflection of the heterogeneous insulin resistance patterns in CKM patients.

More importantly, these novel biomarkers complement the existing AHA staging criteria. Combined with HbA1c and eGFR, they play a more significant role. This synchronous evaluation of multiple pathological processes is a critical gap in current CKM clinical practice that urgently needs to be addressed.

The innovation of this study lies in the first systematic validation of these combined biomarkers’ predictive value across the entire spectrum of CKM-related diseases. Using the large sample data from the National Health and Nutrition Examination Survey (NHANES), we not only confirm their correlation with traditional indicators but also reveal the incremental prognostic information they provide beyond the existing staging system. This will offer essential evidence to support the future updates of CKM diagnostic and treatment guidelines.

The A-H panels in the Supplementary Figure S1 display the ROC curves for two sets of biomarkers, with the left side corresponding to traditional biomarkers and the right side corresponding to the newly proposed biomarkers. The AUC values (95% CI) for each biomarker are shown in the figure, reflecting their discriminative ability. It is evident that there is a significant difference in diagnostic accuracy between the old and new biomarkers for the outcome. This comparison indicates that the clinical relevance of the new biomarkers is acceptable.

## Methods

### Data source and study population

The National Health and Nutrition Examination Survey (NHANES) is a nationally representative cross-sectional study designed to assess the health and nutritional status of the civilian, non-institutionalized population in the United States, using a complex, stratified, multistage probability sampling method. The study adheres to the ethical principles of the Declaration of Helsinki and was approved by the Ethics Review Board of the National Center for Health Statistics. Written informed consent was obtained from all participants. Complete details of the NHANES study design and data are publicly available at www.cdc.gov/nchs/nhanes/. This study analyzed data from six NHANES cycles spanning 1999 to 2018.

Participants were excluded based on the following criteria: lack of data required to define cardiovascular-kidney-metabolic (CKM) syndrome (*n* = 80,978); age <20 years or current pregnancy (*n* = 454). After applying these exclusion criteria, the final analytical sample included 19,884 adults aged ≥20 years (Supplementary Figure S2).

### Variables

#### Metabolism-associated markers

During the research process, we included the following indicators for analysis, and here are their calculation formulas:

1. RAR (Red Cell Distribution Width to Albumin Ratio):

$$RAR=RDW\;\left(\%\right)/\mathrm{ALB}\;\left(g/\mathrm{dL}\right)$$

2. NPAR (Neutrophil Percentage to Albumin Ratio):

$$\mathrm{NPAR}=NCP\;\left(\%\right)/\mathrm{ALB}\;\left(g/\mathrm{dL}\right)$$

3. SIRI (Systemic Immune-Inflammation Index):

$$\begin{array}{l} \mathrm{SIRI}=NC\;\left(1,000\;\mathrm{cells}/\mathrm{\mu L}\right)\times MCC\;\left(1,000\;\mathrm{cells}/\mathrm{\mu L}\right)/\\ LCC\;\left(1,000\;\mathrm{cells}/\mathrm{\mu L}\right) \end{array}$$

4. Homair (Homeostatic Model Assessment for Insulin Resistance):

$$\mathrm{Homair}=\mathrm{FIN}\;\left(\mathrm{mU}/L\right)\times FBG\;\left(\mathrm{mmol}/L\right)/22.5$$

5. eGFR (14) (Estimated Glomerular Filtration Rate):

$$\begin{array}{l} GFR=175\times \mathrm{Standardized}\;{Scr}^{-1.154}\times {\mathrm{Age}}^{-0.203}\times \\ 1.212\;\left(\mathrm{if black}\right)\times 0.742\;\left(\mathrm{if female}\right) \end{array}$$

To validate the relationship between RAR, NPAR, SIRI, and Homair with CKM while excluding other influencing factors, we performed quartile grouping for these four biomarkers. Specifically, the data were divided into four groups based on the weighted values of each factor, with each group containing 25% of the data points. The quartile cutoffs for each factor were determined based on the 25th, 50th, and 75th percentiles, ensuring clear and consistent grouping.

### Definition of outcome variables

In this study, cardiovascular disease (CVD) was defined based on self-reported conditions in NHANES participants, including. Covariates included diabetes mellitus (DM), hypertension (HBP), and chronic kidney disease (CKD), defined by clinical history, medication use, or laboratory criteria (Supplementary Table S1). Cardiovascular-kidney-metabolic [CKM (15)] syndrome was categorized into stages (0 to 4) based on the presence of risk factors, metabolic abnormalities, CKD, and cardiovascular damage or events (Table 1). Detailed definitions and grouping criteria are provided in Supplementary Table S2.

**Table 1:** CKM syndrome staging definitions and clinical significance.

Stage	Core definition	Clinical significance
0	No risk factors	Baseline health reference
1	Adiposity dysfunction only	Early metabolic dysregulation
2	Metabolic risk factors or moderate CKD	Intermediate-risk population
3	High CVD risk or advanced CKD	Preclinical organ damage
4	Established CVD	Highest event risk

### Data collection

Based on our research, we have collected relevant demographic data, physical examinations, laboratory tests, lifestyle habits, and medical conditions from NHANES.

#### Demographics

RACE, EDU (education) (16), GENDER, AGE, MARRY, PIR (Poverty Income Ratio), SOMKING (17), DRINKING (18), SPORT (19).

#### Examinations

FINS (Fasting Insulin, uU/mL), ALB (Albumin, g/dL), UA (Uric Acid, mg/dL), CR (Creatinine, mg/dL), FBG (Fasting Blood Glucose, mg/dL), HbA1c (Hemoglobin A1c, %), WBC (White Blood Cell Count, 10^3^ cells/μL), NCP (Neutrophil Count Percentage, %), NC (Neutrophil Count, 10^3^ cells/μL), RDW (Red Cell Distribution Width, %), TC (Total Cholesterol, mg/dL), TG (Triglycerides, mg/dL), WC (Waist Circumference, cm), Egfr (Estimated Glomerular Filtration Rate), LYC (Lymphocyte Count, 10^3^ cells/μL), MCC (Monocyte Count, 10^3^ cells/μL), PLT (Platelet Count, 10^3^ cells/μL), BMI (Body Mass Index, kg/m^2^), UACR (20) (Urine Albumin-to-Creatinine Ratio, mg/g). Questionnaires: Use of antihypertensive medications, use of antidiabetic medications, use of insulin, use of statins (Supplementary Table S1).

### Ascertainment of mortality

To ascertain the mortality status of the follow-up population, we utilized the NHANES Public Use Linked Mortality File, with data current through December 31, 2019. Mortality follow-up information in NHANES, coded according to the International Classification of Diseases, 10th Edition (ICD-10), is accessible via the Public Use Linked Mortality File. The primary causes of death were classified based on ICD-10 codes. We evaluated all-cause mortality, as well as mortality attributed to cardiovascular diseases, such as Diseases of the heart (I00–I09, I11, I13, I20–I51), Nephritis, nephrotic syndrome, and nephrosis (N00–N07, N17–N19, N25–N27), Diabetes mellitus (E10–E14), and Cerebrovascular diseases (I60–I69).

Detailed methodologies for measuring these variables are publicly available on the NHANES website: www.cdc.gov/nchs/nhanes/.

### Statistical analysis

All statistical analyses were performed using R software (version 4.2.2) and Python (version 3.12.7). Continuous variables were analyzed based on the normality results from the Shapiro–Wilk test. The data were weighted according to the NHANES guidelines and are presented as means with standard errors. Categorical variables are presented as frequencies with percentages.

For baseline characteristics and univariate analysis, comparisons were made according to CKM staging. Normally distributed variables were analyzed using ANOVA, while non-normally distributed variables were analyzed using the Kruskal-Wallis test. Categorical variables were analyzed using the chi-square test or Fisher’s exact test. In univariate logistic regression (stepwise method), variables with *p* < 0.05 were considered potential covariates for subsequent analyses.

For multivariate logistic regression, three nested models were used to analyze four biomarkers (RAR, NPAR, SIRI, Homair). Model 1 was unadjusted; Model 2 adjusted for demographic covariates (age, sex, and race); and Model 3 further adjusted for clinical/metabolic factors (HbA1c, eGFR, UACR, BMI, smoking status). Odds ratios (OR) and 95% confidence intervals (CI) were calculated. The dose–response relationship was evaluated using quartile classification.

To explore the nonlinear relationship between biomarkers and CKM risk, a restricted cubic spline (RCS) analysis with four knots was performed. The optimal cutoff values were determined using the RCS curve based on Model 3, and these values were subsequently used for binary logistic regression.

Survival analysis was conducted using Kaplan–Meier curves and log-rank tests to evaluate the relationship between RAR quartiles and all-cause/cardiovascular/kidney disease mortality. Cox proportional hazards models were used to calculate hazard ratios (HR), and the proportional hazards assumption was tested using Schoenfeld residuals.

To ensure reproducibility, a fixed random seed (random_state = 42) was set for all steps involving randomness, including data splitting and model training. For the train-test split, we categorized CKM stages 0–3 as 0 and CKM stage 4 as 1, using stratified sampling (stratify = y) to maintain consistent distribution across both sets, with a 70:30 ratio. Hyperparameter tuning was performed with grid search (GridSearchCV) for each model’s predefined search space.

Feature selection was conducted using LASSO regression with 10-fold cross-validation (1-SE criterion), resulting in 14 key predictive variables. The model was developed by training 15 algorithms, including XGBoost, LightGBM, and neural networks, on the 70:30 train-test split, with hyperparameters optimized via grid search. Model performance was evaluated using AUC, accuracy, recall, precision, RMSE, and MAE. To enhance model interpretability, SHAP values and partial dependence plots were utilized. Ensemble strategies, including weighted averaging and stacking, were applied to optimize AUC and recall.

Clinical applicability was evaluated using decision curve analysis (DCA), quantifying the net benefit of the prediction model across a range of threshold probabilities (0.08–0.91). Calibration curves were used to assess the consistency between predicted and observed risks.

Sensitivity and subgroup analyses were performed by testing interaction terms between RAR and covariates (e.g., diabetes, hypertension) in stratified logistic regression models. Multiple imputations by chained equations (MICE) were compared with the missing data handling method in LightGBM to assess the robustness of the results.

Software packages used include glmnet (LASSO), rms (RCS), xgboost, lightgbm, scikit-learn (machine learning), survminer (survival analysis), and shap (SHAP visualization). A two-sided *p*-value of < 0.05 was considered statistically significant.

## Results

### Baseline characteristics

Based on the disease staging of CKM, participants’ basic information and clinical characteristics were grouped and statistically analyzed. The study included a total of 19,884 participants, among which 1,881 participants (9.46%) had CKM stage 0, 2,666 participants (13.41%) had stage 1, 11,712 participants (58.90%) had stage 2, 1,298 participants (6.53%) had stage 3, and 2,327 participants (11.70%) had stage 4. The differences in FINS, ALB, UA, CR, FBG, HbA1c, WBC, NCP, NC, RDW, RAR, NPAR, TC, TG, WC, AGE, MARRY, PIR, Egfr, UACR, LYC, MCC, SIRI, Homair, GENDER, RACE, and EDU among the five groups were statistically considerable (*p* < 0.05), while the difference in PLT was not meaningful (*p* > 0.05) (Table 2). After confirming that RAR, NPAR, SIRI, and Homair were all associated with CKM, we further performed univariate logistic regression analysis using DM (21) and Heart Failure (22) as grouping factors, which confirmed that these associations were indeed statistically significant (*p* < 0.05) (Supplementary Tables S3, S4).

**Table 2:** The baseline characteristics of NHANES 1999–2018 participants, stratified by CKM syndrome stages 0–4, weighted for representativeness.

Variable	Total (*n* = 19,884)	0 (*n* = 1881)	1 (*n* = 2,666)	2 (*n* = 11,712)	3 (*n* = 1,298)	4 (*n* = 2,327)	Statistic	*P*
FINS, Mean (SE)	12.78 (0.14)	5.97 (0.14)	11.12 (0.25)	14.36 (0.19)	10.32 (0.42)	15.60 (0.44)	*F* = 356.30	<0.001
ALB, Mean (SE)	4.25 (0.01)	4.36 (0.01)	4.26 (0.01)	4.26 (0.01)	4.21 (0.01)	4.07 (0.01)	*F* = 435.94	<0.001
UA, Mean (SE)	329.37 (0.87)	277.81 (2.07)	316.35 (2.22)	340.36 (1.06)	317.77 (2.94)	352.39 (2.76)	*F* = 356.32	<0.001
CR, Mean (SE)	77.98 (0.31)	71.92 (0.43)	75.12 (0.41)	76.46 (0.28)	88.47 (2.75)	91.61 (1.49)	*F* = 165.02	<0.001
FBG, Mean (SE)	106.61 (0.29)	91.99 (0.33)	104.53 (0.22)	109.34 (0.42)	98.42 (0.77)	116.79 (1.02)	*F* = 415.79	<0.001
HbA1c, Mean (SE)	5.63 (0.01)	5.21 (0.01)	5.45 (0.01)	5.71 (0.01)	5.40 (0.02)	6.04 (0.03)	*F* = 586.48	<0.001
WBC, Mean (SE)	6.83 (0.03)	6.11 (0.06)	6.64 (0.05)	6.97 (0.03)	6.79 (0.08)	7.16 (0.06)	*F* = 186.74	<0.001
NCP, Mean (SE)	57.98 (0.11)	56.32 (0.30)	57.02 (0.23)	58.20 (0.12)	58.27 (0.29)	59.88 (0.26)	*F* = 96.69	<0.001
NC, Mean (SE)	4.03 (0.02)	3.51 (0.04)	3.85 (0.03)	4.12 (0.02)	4.02 (0.06)	4.33 (0.04)	*F* = 191.42	<0.001
RDW, Mean (SE)	13.08 (0.02)	12.88 (0.04)	13.13 (0.03)	12.96 (0.02)	13.25 (0.03)	13.90 (0.06)	*F* = 193.18	<0.001
RAR, Mean (SE)	3.11 (0.01)	2.98 (0.01)	3.10 (0.01)	3.07 (0.01)	3.17 (0.01)	3.47 (0.03)	*F* = 388.47	<0.001
NPAR, Mean (SE)	13.74 (0.03)	13.01 (0.09)	13.48 (0.06)	13.75 (0.03)	13.89 (0.08)	14.89 (0.09)	*F* = 250.37	<0.001
PLT, Mean (SE)	249.56 (0.80)	240.80 (1.84)	243.06 (1.63)	255.79 (0.87)	237.33 (1.93)	240.58 (2.29)	*F* = 0.48	0.491
TC, Mean (SE)	5.07 (0.01)	4.63 (0.03)	4.86 (0.02)	5.29 (0.02)	4.74 (0.04)	4.76 (0.03)	*F* = 25.27	<0.001
TG, Mean (SE)	136.41 (1.26)	70.96 (0.78)	83.24 (0.71)	167.35 (1.85)	86.85 (1.45)	141.82 (2.90)	*F* = 614.97	<0.001
WC, Mean (SE)	99.07 (0.22)	78.98 (0.19)	96.83 (0.45)	102.34 (0.22)	100.06 (0.51)	106.57 (0.55)	*F* = 1943.84	<0.001
AGE, Mean (SE)	48.35 (0.22)	36.99 (0.47)	44.76 (0.37)	49.84 (0.22)	42.90 (0.67)	62.52 (0.54)	*F* = 1151.55	<0.001
PIR, Mean (SE)	2.96 (0.03)	3.11 (0.06)	3.03 (0.05)	3.00 (0.03)	2.85 (0.08)	2.54 (0.06)	*F* = 44.57	<0.001
Egfr, Mean (SE)	95.99 (0.35)	107.75 (0.72)	102.21 (0.66)	95.76 (0.37)	94.03 (1.24)	78.12 (0.67)	*F* = 955.84	<0.001
UACR, Mean (SE)	87.59 (10.30)	31.51 (5.07)	13.54 (2.01)	80.56 (8.43)	199.24 (80.34)	224.52 (50.54)	*F* = 19.54	<0.001
LYC, Mean (SE)	2.01 (0.01)	1.88 (0.02)	2.01 (0.02)	2.05 (0.01)	2.00 (0.02)	1.96 (0.03)	*F* = 6.49	0.012
MCC, Mean (SE)	0.54 (0.00)	0.50 (0.01)	0.53 (0.00)	0.55 (0.00)	0.53 (0.01)	0.59 (0.01)	*F* = 145.06	<0.001
SIRI, Mean (SE)	1.21 (0.01)	1.02 (0.02)	1.14 (0.02)	1.22 (0.01)	1.17 (0.02)	1.54 (0.03)	*F* = 195.82	<0.001
Homair, Mean (SE)	65.01 (0.86)	24.88 (0.65)	52.66 (1.28)	74.21 (1.22)	47.55 (2.49)	88.81 (3.96)	*F* = 266.05	<0.001
GENDER, *n* (%)							χ^2^ = 184.39	<0.001
Male	10,013 (49.93)	777 (38.83)	1,344 (50.96)	6,139 (52.84)	540 (42.21)	1,213 (49.49)		
Female	9,871 (50.07)	1,104 (61.17)	1,322 (49.04)	5,573 (47.16)	758 (57.79)	1,114 (50.51)		
RACE, *n* (%)							χ^2^ = 196.99	<0.001
1	3,418 (8.01)	189 (5.14)	514 (10.68)	2,234 (8.14)	202 (9.94)	279 (5.08)		
2	1718 (5.48)	146 (5.55)	273 (6.01)	1,001 (5.30)	132 (7.93)	166 (3.92)		
3	8,860 (68.15)	811 (68.69)	1,025 (64.36)	5,249 (68.93)	558 (64.61)	1,217 (71.08)		
4	3,917 (11.12)	342 (9.99)	505 (10.64)	2,239 (10.77)	297 (13.07)	534 (13.97)		
5	1971 (7.24)	393 (10.63)	349 (8.31)	989 (6.86)	109 (4.45)	131 (5.95)		
EDU, *n* (%)							χ^2^ = 415.86	<0.001
1	2,510 (6.47)	103 (3.16)	267 (5.30)	1,609 (6.80)	129 (5.43)	402 (11.10)		
2	2,934 (11.55)	216 (8.58)	392 (10.57)	1771 (12.04)	140 (8.11)	415 (16.17)		
3	4,579 (24.32)	374 (19.72)	597 (23.52)	2,789 (25.27)	270 (22.51)	549 (26.64)		
4	5,540 (30.50)	564 (30.67)	743 (28.84)	3,214 (31.04)	436 (33.81)	583 (27.04)		
5	4,294 (27.16)	624 (37.88)	665 (31.77)	2,313 (24.86)	321 (30.14)	371 (19.06)		
SMOKING, *n* (%)							χ^2^ = 311.64	<0.001
0	10,613 (52.86)	1,196 (61.22)	1,535 (56.01)	6,083 (51.27)	792 (61.21)	1,007 (41.50)		
1	5,205 (26.12)	257 (15.88)	606 (24.79)	3,200 (27.25)	278 (22.09)	864 (36.65)		
2	4,045 (21.02)	426 (22.90)	522 (19.20)	2,416 (21.48)	227 (16.69)	454 (21.85)		
DRINKING, *n* (%)							χ^2^ = 80.32	<0.001
0	4,853 (25.18)	329 (20.84)	495 (22.87)	3,142 (25.71)	211 (20.75)	676 (31.51)		
1	11,513 (74.82)	965 (79.16)	1,330 (77.13)	7,302 (74.29)	623 (79.25)	1,293 (68.49)		
SPORT, *n* (%)							χ^2^ = 326.52	<0.001
1	6,516 (34.32)	483 (23.67)	822 (30.60)	3,845 (36.38)	392 (27.77)	974 (47.60)		
2	10,505 (65.68)	1,242 (76.33)	1,668 (69.40)	5,854 (63.62)	816 (72.23)	925 (52.40)		
CKD, *n* (%)							χ^2^ = 1214.64	<0.001
0	15,228 (93.27)	1,350 (99.10)	1904 (98.89)	9,819 (94.51)	705 (85.39)	1,450 (76.52)		
1	1,493 (6.73)	16 (0.90)	28 (1.11)	726 (5.49)	203 (14.61)	520 (23.48)		
CVD, *n* (%)							χ^2^ = 19884.00	<0.001
0	15,505 (90.51)	1,430 (100.00)	2016 (100.00)	11,128 (100.00)	931 (100.00)	0 (0.00)		
1	2087 (9.49)	0 (0.00)	0 (0.00)	0 (0.00)	0 (0.00)	2087 (100.00)		
HBP, *n* (%)							χ^2^ = 8141.03	<0.001
0	7,307 (46.02)	1,430 (100.00)	2015 (100.00)	2,718 (26.84)	729 (85.87)	415 (22.78)		
1	10,279 (53.98)	0 (0.00)	0 (0.00)	8,409 (73.16)	198 (14.13)	1,672 (77.22)		
DM, *n* (%)							χ^2^ = 6190.19	<0.001
0	5,677 (33.66)	0 (0.00)	326 (13.59)	4,508 (42.37)	780 (89.40)	63 (3.34)		
1	3,060 (13.35)	0 (0.00)	0 (0.00)	2,252 (16.12)	57 (3.91)	751 (31.89)		
2	8,852 (52.99)	1,430 (100.00)	1,690 (86.41)	4,369 (41.51)	90 (6.70)	1,273 (64.77)		
MARRY, *n* (%)							χ^2^ = 1039.01	<0.001
1	10,589 (56.59)	822 (46.38)	1,413 (56.84)	6,518 (59.20)	610 (50.12)	1,226 (57.27)		
2	1792 (6.35)	61 (1.83)	119 (3.45)	1,055 (6.46)	123 (5.96)	434 (15.89)		
3	2064 (10.19)	129 (6.73)	261 (9.87)	1,270 (10.82)	111 (9.69)	293 (11.41)		
4	655 (2.41)	51 (2.30)	85 (2.20)	410 (2.54)	36 (1.96)	73 (2.39)		
5	3,168 (16.66)	629 (32.07)	529 (18.69)	1,544 (14.09)	273 (21.04)	193 (7.50)		
6	1,457 (7.80)	179 (10.70)	252 (8.95)	791 (6.90)	139 (11.23)	96 (5.55)		
BMIQ, *n* (%)							χ^2^ = 6980.87	<0.001
1	332 (1.86)	0 (0.00)	166 (6.48)	123 (1.26)	43 (2.76)	0 (0.00)		
2	5,408 (28.98)	1863 (99.23)	644 (24.92)	2,429 (20.77)	78 (4.17)	394 (17.87)		
3	6,474 (32.38)	18 (0.77)	992 (37.71)	4,064 (34.66)	643 (53.40)	757 (33.45)		
4	7,348 (36.78)	0 (0.00)	833 (30.89)	4,941 (43.31)	495 (39.68)	1,079 (48.68)		

SE: standard error; F: ANOVA, χ^2^: Chi-square test. BMIQ: (1) <18 (Underweight), (2) 18–25 (Normal weight), (3) 25–30 (Overweight), (4) ≥ 30 (Obese). CVD (Cardiovascular Disease), DM (Diabetes Mellitus), HBP (High Blood Pressure), CKD (Chronic Kidney Disease), FINS (Fasting Insulin), ALB (Albumin), UA (Uric Acid), CR (Creatinine), FBG (Fasting Blood Glucose), HbA1c (Hemoglobin A1c), WBC (White Blood Cell Count), NCP (Neutrophil Count Percentage), NC (Neutrophil Count), RDW (Red Cell Distribution Width), TC (Total Cholesterol), TG (Triglycerides), WC (Waist Circumference), eGFR (Estimated Glomerular Filtration Rate), LYC (Lymphocyte Count), MCC (Monocyte Count), PLT (Platelet Count), BMI (Body Mass Index), UACR (Urine Albumin-to-Creatinine Ratio), EDU (Education), PIR (Poverty Income Ratio), RAR (Red Cell Distribution Width to Albumin Ratio), NPAR (Neutrophil Percentage to Albumin Ratio), SIRI (Systemic Immune-Inflammation Index), Homair (Homeostatic Model Assessment for Insulin Resistance), eGFR (Estimated Glomerular Filtration Rate), RCS (Restricted Cubic Splines), AUC (Area Under the ROC Curve).

### Multimodal logistic regression analysis

After baseline data analysis, to validate the relationship between RAR, NPAR, SIRI, and Homair with CKM while excluding other influencing factors, we grouped each biomarker into quartiles based on their weighted values, using the 25th, 50th, and 75th percentiles as cutoffs (Supplementary Table S5) and then conducted multimodal logistic regression analysis (Table 3). The results showed that all variables exhibited a clear dose-dependent effect in the unadjusted model (Model 1), meaning that as the variable levels increased, the odds ratio (OR) markedly increased (*p* < 0.05). In Model 3, after stepwise adjustment for confounding factors, all factors maintained substantial differences (*p* < 0.05), with RAR being the most significant (OR: 2.73, 95% CI: 2.07–3.59, *p* < 0.001), indicating that its association with the outcome was independent of the adjusted confounding factors and may be a key independent predictor. NPARQ, SIRIQ, and HomairQ showed certain changes after adjustment: NPARQ in Model 3 had an OR of 1.41 (1.13–1.77, *p* = 0.003); although the effect size decreased, it remained robust. SIRIQ in Model 3 had an OR of 1.47 (1.15–1.88, *p* = 0.003), also showing a decreased effect size but still significant after adjustment. HomairQ in Model 3 had an OR of 1.52 (1.11–2.07, *p* = 0.009), with the adjusted effect size decreasing but still meaningful.

**Table 3:** Multimodal logistic regression analysis of four biomarkers, weighted for representativeness.

Variables	Model 1		Model 2		Model 3	
	OR (95%CI)	*P*	OR (95%CI)	*P*	OR (95%CI)	*P*
NPARQ						
1	1.00 (Reference)		1.00 (Reference)		1.00 (Reference)	
2	1.23 (1.03 ~ 1.48)	0.026	1.14 (0.93 ~ 1.39)	0.203	1.05 (0.80 ~ 1.37)	0.73
3	1.59 (1.32 ~ 1.92)	<0.001	1.28 (1.04 ~ 1.58)	0.021	1.04 (0.79 ~ 1.35)	0.8
4	2.69 (2.26 ~ 3.20)	<0.001	1.91 (1.59 ~ 2.30)	<0.001	1.41 (1.13 ~ 1.77)	0.003
SIRIQ						
1	1.00 (Reference)		1.00 (Reference)		1.00 (Reference)	
2	1.21 (1.01 ~ 1.46)	0.049	1.11 (0.90 ~ 1.37)	0.343	1.08 (0.84 ~ 1.41)	0.542
3	1.53 (1.28 ~ 1.83)	<0.001	1.28 (1.04 ~ 1.57)	0.022	1.19 (0.92 ~ 1.53)	0.187
4	2.69 (2.30 ~ 3.16)	<0.001	1.84 (1.54 ~ 2.20)	<0.001	1.47 (1.15 ~ 1.88)	0.003
RARQ						
1	1.00 (Reference)		1.00 (Reference)		1.00 (Reference)	
2	1.37 (1.09 ~ 1.72)	0.007	1.02 (0.81 ~ 1.28)	0.882	1.15 (0.86 ~ 1.55)	0.34
3	2.05 (1.74 ~ 2.43)	<0.001	1.37 (1.15 ~ 1.63)	<0.001	1.57 (1.23 ~ 2.00)	<0.001
4	4.33 (3.62 ~ 5.18)	<0.001	2.53 (2.05 ~ 3.13)	<0.001	2.73 (2.07 ~ 3.59)	<0.001
HomairQ						
1	1.00 (Reference)		1.00 (Reference)		1.00 (Reference)	
2	1.33 (1.11 ~ 1.59)	0.002	1.05 (0.85 ~ 1.29)	0.660	1.08 (0.83 ~ 1.40)	0.577
3	1.71 (1.42 ~ 2.06)	<0.001	1.18 (0.95 ~ 1.46)	0.131	1.33 (1.00 ~ 1.75)	0.051
4	2.46 (2.03 ~ 2.98)	<0.001	1.38 (1.10 ~ 1.71)	0.005	1.52 (1.11 ~ 2.07)	0.009

OR: odds ratio, CI: confidence interval.

Model 1: Crude.

Model 2: Adjust: GENDER, RACE, EDU, AGEQ, MARRY, PIR.

Model 3: Adjust: GENDER, RACE, EDU, SMOKING, DRINKING, SPORT, HBP, DM, AGEQ, BMI, WBC, PIR.

RAR (Red Cell Distribution Width to Albumin Ratio), NPAR (Neutrophil Percentage to Albumin Ratio), SIRI (Systemic Immune-Inflammation Index), Homair (Homeostatic Model Assessment for Insulin Resistance).

HomairQ (P25 = 26.1, P50 = 43.44, P75 = 74.389), SIRIQ (P25 = 0.69, P50 = 1, P75 = 1.467), NPARQ (P25 = 12.045, P50 = 13.659, P75 = 15.311), and RARQ (P25 = 2.804, P50 = 3.023, P75 = 3.293).

In summary, RARQ remained prominent with a high effect size in all adjusted models, suggesting that it may be a strong independent predictor. However, NPARQ, SIRIQ, and HomairQ, although showing a decrease in effect size after adjustment, still remained relevant, indicating that the associations between these variables and the outcome are somewhat independent of confounding factors but may still be influenced by some confounding factors. We observed that the effect of RAR on CKM was the largest among the indicators studied. To further investigate the relationship between RAR and other factors, we grouped participants by quartiles of RAR and performed univariate logistic regression analysis to examine its relationship with other variables (Supplementary Table S6).

### RCS and binary logistic regression

**Figure 1:**
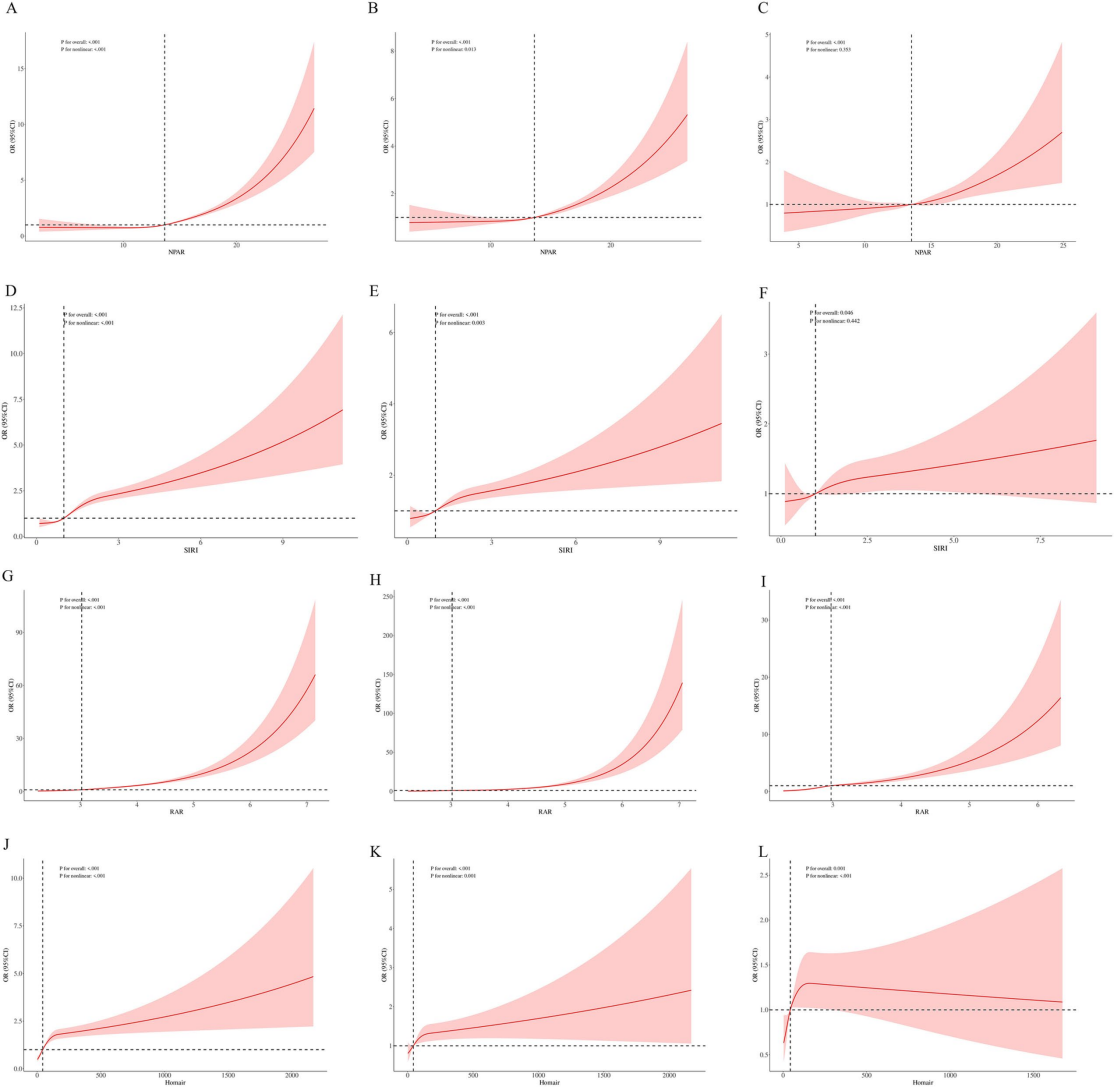
Restricted cubic spline. Based on the results from multiple logistic regression models, we used restricted cubic spline models to examine the association between the presence of CKD and four biomarkers in the NHANES (1999–2018) dataset. The odds ratio (OR) (red line) and 95% confidence interval (shaded area) were calculated. **(A–C)** RCS Curve for NPAR and CKM. **(D–F)** RCS Curve for SIRI and CKM. **(G–I)** RCS Curve for RAR and CKM. **(J–L)** RCS Curve for HMOAIR and CKM. Model 1: **(A,D,G,J)**. Adjust: Crude. Model 2: **(B,E,H,K)**. Adjust: GENDER, RACE, EDU, AGEQ, MARRY, PIR. Model 3: **(C,F,I,L)**. Adjust: GENDER, RACE, EDU, SMOKING, DRINKING, SPORT, HBP, DM, AGEQ, BMI, MARRY, WBC, PIR.

Based on the multimodal logistic regression analysis, we plotted the restricted cubic spline (RCS) curves for the four biomarkers across three models. RAR demonstrated statistical differences in the RCS of all three models, with a nonlinear correlation (P for nonlinear: <0.001), indicating that the relationship between RAR and CKM is not linear, but rather nonlinear (shown in Figures 1G–I). Homair also showed statistical differences in all three models (P for overall < 0.05), and the correlation between them was nonlinear (P for nonlinear: <0.001), as seen in Figures 1J–L. NPAR exhibited a nonlinear relationship in Model 1 (Figure 1A) and Model 2 (Figure 1B) (P for nonlinear: <0.05), but in Model 3 (Figure 1C), the relationship with CKM became linear after adjusting for several covariates. Differences were statistically significant in all three models (P for overall <0.001). SIRI displayed a similar pattern, with statistical differences between groups in Model 1 (Figure 1D), Model 2 (Figure 1E), and Model 3 (Figure 1F) (P for overall < 0.05). In Models 1 and 3, SIRI showed a nonlinear correlation with CKM (P for nonlinear: <0.05), whereas Model 2 indicated a linear relationship (P for nonlinear: >0.05).

We determined the optimal cutoff based on the RCS in Model 3 and then performed a binary logistic regression analysis (Supplementary Table S7). The results showed that RAR, NPAR, and Homair had statistically significant differences between the two groups (*p* < 0.05). However, for SIRI in Node 1, although the OR = 0.52, the *p*-value was 0.438, indicating that this association was not statistically significant, suggesting that the effect of SIRI at this node was weak. In contrast, in Node 2, SIRI was significantly associated with the outcome (OR = 1.92, *p* < 0.001), indicating that higher SIRI values were associated with the occurrence of the outcome, and this relationship was statistically significant.

### Cox proportional hazards regression and Kaplan–Meier survival curves

We performed multimodal Cox proportional hazards regression analysis on these four biomarkers based on survival outcomes and survival time. The results showed that RAR, SIRI, and NPAR remained statistically significant in the fourth quartile after adjusting for factors such as GENDER, RACE, EDU, SMOKING, DRINKING, SPORT, HBP, DM, AGEQ, BMI, MARRY, WBC, and PIR (*p* < 0.001), indicating a notable relationship with survival outcomes (Table 4).

**Table 4:** Multi-model Cox regression of four biomarkers, weighted for representativeness.

Variables	Model1		Model2		Model3	
	HR (95%CI)	*P*	HR (95%CI)	*P*	HR (95%CI)	*P*
SIRIQ						
1	1.00 (Reference)		1.00 (Reference)		1.00 (Reference)	
2	1.23 (1.05–1.43)	0.011	1.02 (0.87–1.19)	0.846	0.92 (0.74–1.16)	0.481
3	1.81 (1.55–2.12)	<0.001	1.31 (1.12–1.55)	0.001	1.21 (0.98–1.50)	0.074
4	3.21 (2.78–3.69)	<0.001	1.75 (1.50–2.04)	<0.001	1.44 (1.19–1.75)	<0.001
RARQ						
1	1.00 (Reference)		1.00 (Reference)		1.00 (Reference)	
2	1.87 (1.62–2.15)	<0.001	1.28 (1.12–1.47)	<0.001	1.24 (1.04–1.47)	0.014
3	2.69 (2.35–3.08)	<0.001	1.48 (1.30–1.69)	<0.001	1.36 (1.13–1.64)	0.001
4	4.84 (4.21–5.56)	<0.001	2.55 (2.24–2.90)	<0.001	2.38 (1.98–2.88)	<0.001
NPARQ						
1	1.00 (Reference)		1.00 (Reference)		1.00 (Reference)	
2	1.33 (1.13–1.57)	<0.001	1.15 (0.96–1.38)	0.120	1.10 (0.87–1.38)	0.436
3	1.63 (1.40–1.90)	<0.001	1.20 (1.04–1.39)	0.014	1.13 (0.93–1.36)	0.211
4	3.03 (2.64–3.48)	<0.001	1.81 (1.58–2.08)	<0.001	1.65 (1.37–1.98)	<0.001
HomairQ						
1	1.00 (Reference)		1.00 (Reference)		1.00 (Reference)	
2	1.10 (0.97–1.24)	0.152	0.91 (0.80–1.04)	0.161	0.90 (0.76–1.08)	0.265
3	1.09 (0.97–1.24)	0.155	0.87 (0.75–1.01)	0.070	0.92 (0.76–1.11)	0.407
4	1.47 (1.27–1.69)	<0.001	1.18 (1.01–1.39)	0.037	0.93 (0.76–1.14)	0.478

HR: hazard ratio, CI: confidence interval.

Model 1: Crude.

Model 2: Adjust: GENDER, RACE, EDU, AGEQ, MARRY, PIR.

Model 3: Adjust: GENDER, RACE, EDU, SMOKING, DRINKING, SPORT, HBP, DM, AGEQ, BMI, MARRY, WBC, PIR.

HomairQ (P25 = 26.1, P50 = 43.44, P75 = 74.389), SIRIQ (P25 = 0.69, P50 = 1, P75 = 1.467), NPARQ (P25 = 12.045, P50 = 13.659, P75 = 15.311), and RARQ (P25 = 2.804, P50 = 3.023, P75 = 3.293).

**Figure 2:**
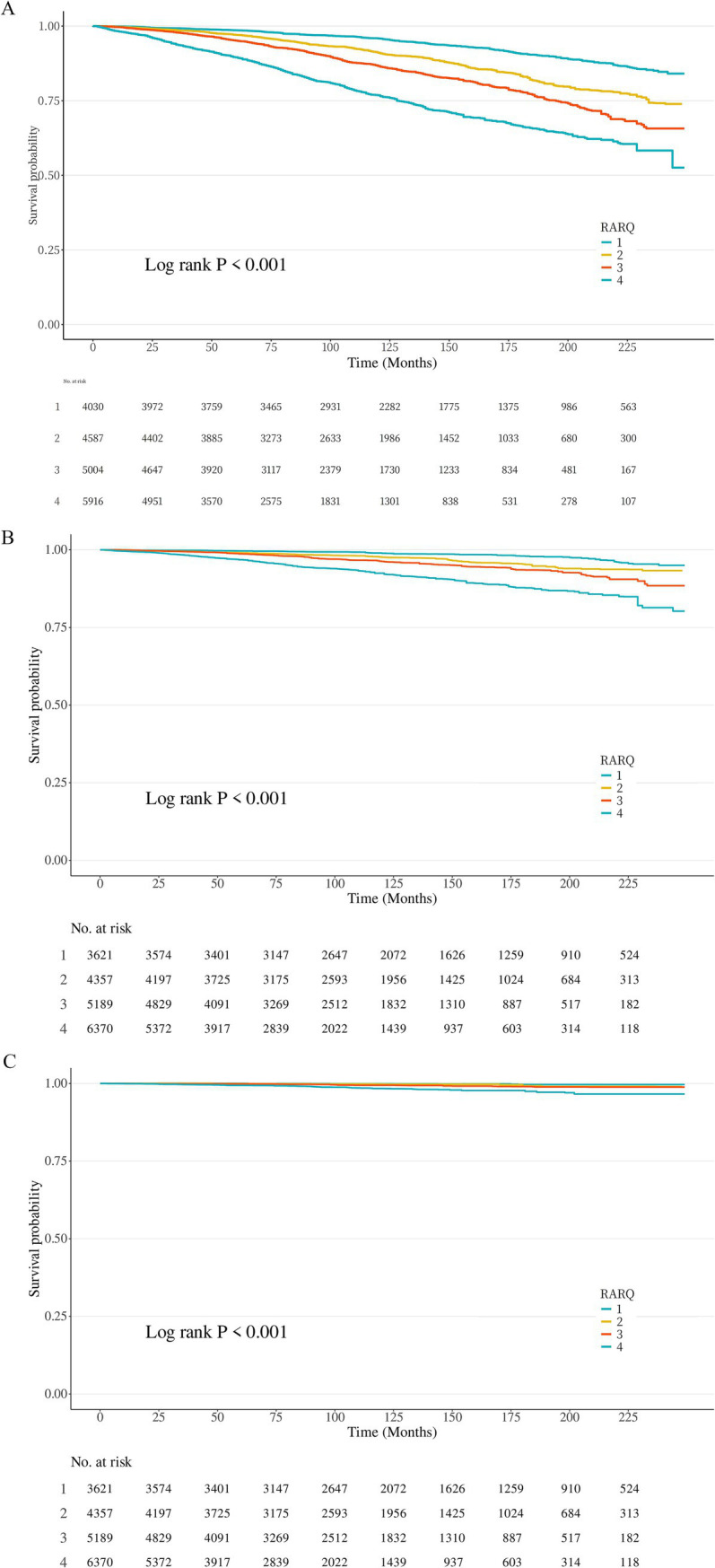
Kaplan–Meier survival curves by RAR groups. Kaplan–Meier survival curves for all-cause mortality **(A)**, cardiovascular disease mortality **(B)**, and kidney disease mortality **(C)** by RARQ groups. RARQ group: 1 (Blue), 2 (Orange), 3 (Red), 4 (Green). **(A)** All-cause mortality. **(B)** cardiovascular disease mortality. **(C)** Kidney disease mortality.

Based on these statistical results, RAR exhibited a significant predictive role for CKM. Therefore, we further conducted survival analysis for RAR, including survival curves for all-cause mortality, cardiovascular disease mortality, and kidney disease mortality (Figure 2). The results showed that as RAR quartiles increased (i.e., as risk values increased), survival probability gradually decreased, indicating that individuals with higher RAR values had lower survival rates during the study period. Specifically, the survival curve analysis for RAR and all-cause mortality (Log-rank *p* < 0.001) (Figure 2A) and cardiovascular disease mortality (Log-rank *p* < 0.001) (Figure 2B) demonstrated that the mortality rate in the fourth quartile of RAR was substantially higher than in the other groups. Although the survival curve for kidney disease mortality did not show clear graphical differences, statistical analysis revealed a significant difference (Log-rank *p* < 0.001) (Figure 2C). These findings highlight that RAR is a strong risk factor associated with mortality risk, and there is a notable association between RAR quartiles and survival rates. This further underscores the importance of RAR as a survival prediction factor.

### Machine learning model selection

To further validate the role of various inflammation-nutritional-metabolic indicators in this dataset, we performed machine learning analysis to confirm their predictive value by studying their importance and interactions. First, we conducted LASSO analysis and selected 14 variables based on the LASSO path plot (Supplementary Figure S3A) and cross-validation error curve (Supplementary Figure S3B) using the 1se criterion (Supplementary Figure S3C). Subsequently, we used these variables for machine learning analysis.

**Figure 3:**
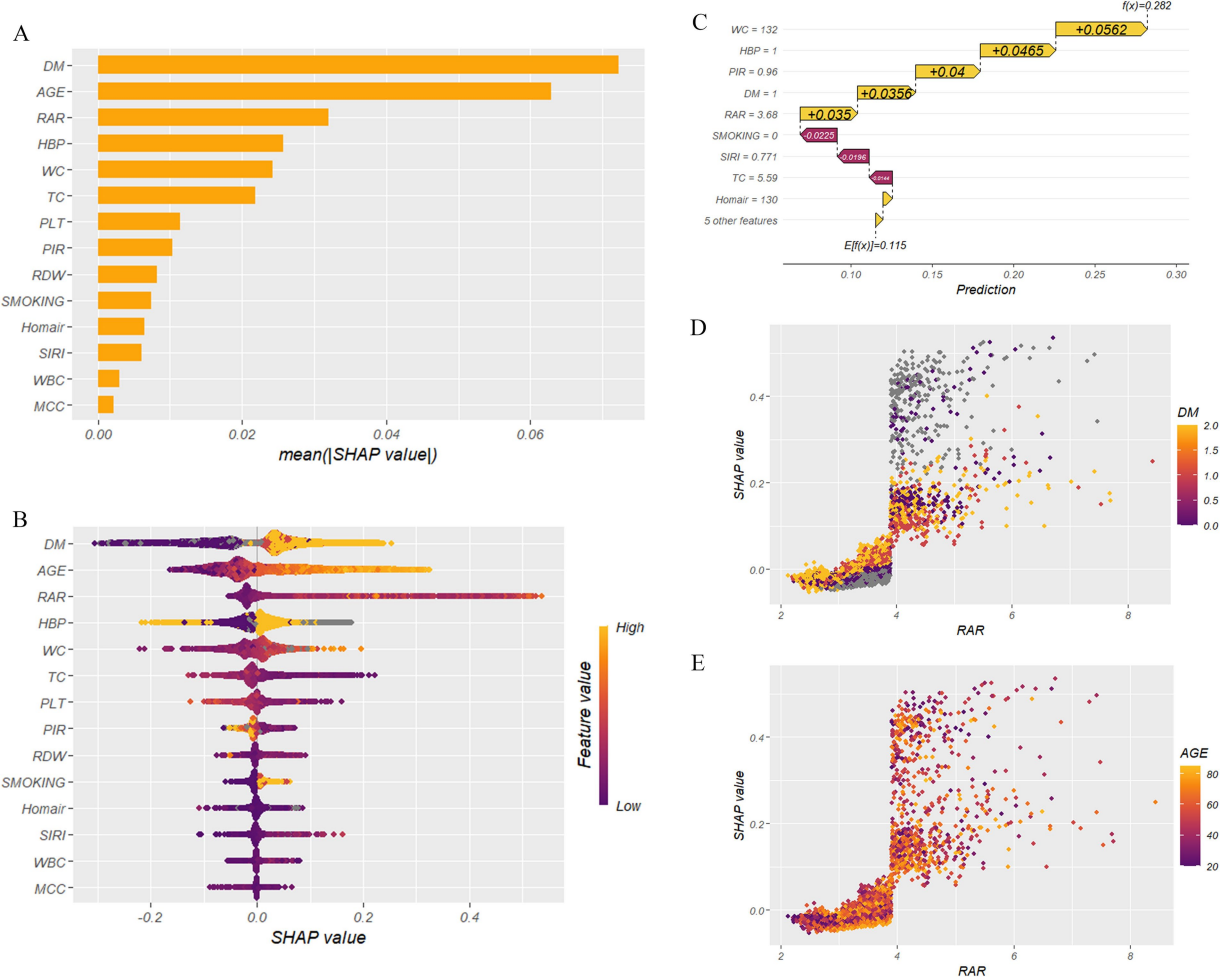
SHAP analysis of machine learning. **(A)** Importance of the SHAP representation of CKM. **(B)** Bee warm SHAP analysis of CKM. **(C)** Waterfall plot of SHAP analysis of CKM. **(D)** SHAP dependence plot of RAR-DM for CKM. **(E)** SHAP dependence plot of RAR-AGE for CKM. DM (diabetes mellitus), HBP (high blood pressure), WBC (white blood cell count), TC (total cholesterol), WC (waist circumference), MCC (monocyte count), PLT (platelet count), PIR (poverty income ratio), RAR (red cell distribution width to albumin ratio), SIRI (systemic immune-inflammation index), Homair (homeostatic model assessment for insulin resistance).

**Figure 4:**
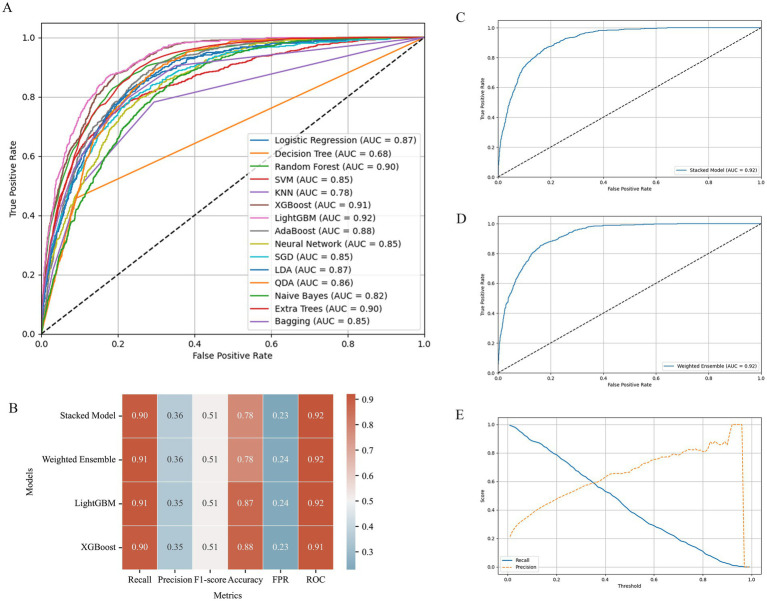
Machine learning model. **(A)** ROC curve comparison of machine learning models. **(B)** Model performance on test set, the color ranges from cyan (low values) to red (high values). **(C)** ROC curve – stacked model. **(D)** ROC curve – weighted ensemble. **(E)** LightGBM-precision & recall vs. threshold.

In the machine learning analysis, we simulated 15 different models (Figure 3A) and compared the *R*^2^, RMSE, and MAE for the test and validation sets (Supplementary Figure S4A). The results showed that models such as XGBoost, LightGBM, and Neural Network did not exhibit overfitting in the training set (Supplementary Figure S4C) and performed well with low error in the test set (Supplementary Figure S4B), indicating strong generalization ability. Next, we calculated the ROC curve and AUC values for each model and, based on the AUC results and the fit, selected LightGBM (AUC = 0.92) and XGBoost (AUC = 0.91) for the construction of a new model through multi-model combination (Figure 4A).

We first built the model using a weighted approach, determining the best 1:1 weighted combination through a grid search, which yielded the highest AUC value (0.92) (Figure 4D). Subsequently, we stacked the two models, also using a grid search to find the best AUC value for the stacked model (0.92) (Figure 4C). Since Recall and Precision cannot increase simultaneously (Figure 4E), we chose Recall as the primary evaluation metric, setting a minimum Recall of 90% for model performance evaluation, in line with the severe prognostic nature of CKM disease. Under this condition, LightGBM demonstrated the best performance (Figure 4B).

Specifically, LightGBM achieved a Recall rate of 91%, which is equal to or higher than other models, effectively reducing the risk of missed diagnoses and ensuring that the majority of affected individuals are correctly identified. Additionally, LightGBM excelled in accuracy, achieving 0.87, indicating its higher precision in classifying healthy versus affected individuals. Meanwhile, LightGBM, as an efficient gradient boosting framework, has a significant training speed advantage on large datasets, enabling rapid response to model updates and prediction tasks. Although its false positive rate was 0.24, this value was relatively close among all models, and LightGBM’s advantages in Recall and accuracy offset this. Therefore, LightGBM is the model that best meets our needs (consistent with other research findings (23)).

### SHAP analysis

We built a predictive model based on the LightGBM algorithm and conducted an analysis. The dataset was split into a training set and a test set in a 7:3 ratio, and we applied ten-fold cross-validation for model training, using the default parameters from the R package. The final prediction results were obtained. Since the dataset contained missing values, although the LightGBM model itself has the ability to handle missing data (missing values are represented in grayscale in SHAP analysis), we decided to use the k-nearest neighbors imputation method (knn = 5) and compare the results with those of the untreated dataset (Supplementary Figure S5).

In the analysis without handling the missing values (Figure 3), the feature importance plot showed that the top three variables were DM, AGE, and RAR (Figure 3A). From the SHAP beeswarm plot (Figure 3B), we can see that the yellowish colors represent larger variable values, with high age, diabetes, and high RAR values showing deep yellow and being distributed in the positive direction of the SHAP values. This indicates that these factors have a positive influence on the CKM outcome. This result was similarly confirmed in the waterfall plot (Figure 3C).

Moreover, in the dependence plots for RAR-AGE (Figure 3E) and RAR-DM (Figure 3D), high RAR values are primarily distributed in the positive direction of the SHAP values, and these regions are also concentrated with individuals who are older or have diabetes. In contrast, low RAR values are mostly distributed in the negative direction of the SHAP values, with these areas concentrating younger individuals or those who do not have diabetes. These results suggest that RAR, age, and diabetes status significantly influence CKM outcome prediction, and higher RAR values are closely associated with adverse outcomes.

### DCA (decision curve analysis)

**Figure 5:**
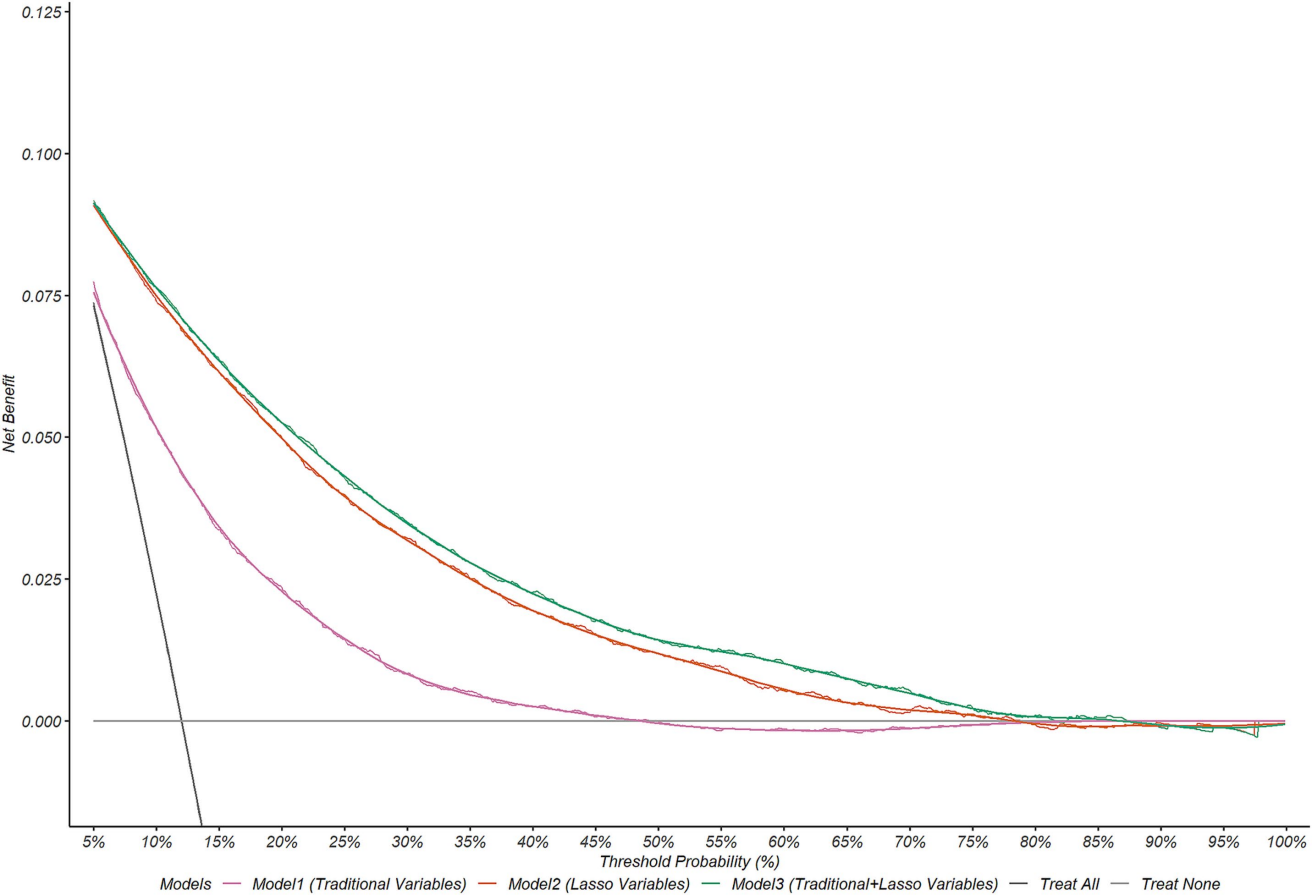
Decision curve analysis.

Based on the variables selected by Lasso and kidney metabolic indicators (HbA1c and eGFR), these were incorporated into the decision curve analysis (DCA) (Figure 5) and subsequently validated using a calibration curve (Supplementary Figure S6). In this analysis, Model 1 (AUC = 0.741) includes only the traditional variables HbA1c and eGFR, Model 2 (AUC = 0.861) includes all Lasso-selected variables, and Model 3 (AUC = 0.867) includes all of the above indicators. The results show that, compared to the “no intervention” and “complete intervention” scenarios, all three prediction models provided significant net benefit improvement at certain thresholds of CKM (event occurrence), with Model 2 and Model 3 being the most outstanding. Moreover, when the threshold probability for CKM occurrence was between approximately 0.08 and 0.48, Models 3 and 2 significantly outperformed Model 1. When the threshold probability for CKM occurrence was between approximately 0.08 and 0.875, Model 3 showed slightly better overall prediction performance than Model 2, suggesting that within this range, our model has clinical utility, and the combined use of both new and traditional indicators achieves better results. The calibration curve (Supplementary Figure S6) analysis shows that the ideal perfect prediction is represented by the 45-degree dashed line. Among all the models, model3 and model2 have calibration curves that are closest to the dashed line, indicating the best prediction accuracy. In contrast, model1 shows a noticeable deviation from the line, suggesting relatively poorer performance. The proximity of the calibration curve to the dashed line directly reflects model performance: the closer the curve is to the line, the more consistent the model’s predictions are with actual observations. Conversely, the deviation in model1 indicates that this model may be experiencing overfitting or underfitting issues. This calibration analysis underscores the real-world applicability of the models, as the closer they are to the ideal line, the more reliable their predictions for real-world scenarios.

### Subgroup analysis

We conducted a subgroup analysis to explore the interactions between different variables (including GENDER, RACE, education, smoking, drinking, sport, HBP, DM, AGE, and BMI) and RAR, and further investigate the relationship between RAR and CKM. In Supplementary Figure S7, we found that variables such as gender, race, sport, BMI, and drinking did not show significant interactions with RAR (*p* > 0.05). Age: The young group (AGEQ = 1) shows the strongest response to RAR (OR = 6.82), with the relationship weakening in older groups (AGEQ = 3, OR = 2.19). AGE significantly interacts with RAR and CKM (P for interaction < 0.001), indicating that age should be considered in interventions and risk prediction. SMOKING: Smokers show higher OR values, indicating that smoking and alcohol consumption strengthen the RAR-CKM relationship. Smoking significantly moderates this relationship (P for interaction = 0.010). Education level: Lower education levels make individuals more sensitive to RAR (P for interaction = 0.029), suggesting they may be more affected by RAR during CKM progression. HBP: The hypertensive group (OR = 4.42) shows a stronger relationship between RAR and CKM than the non-hypertensive group (OR = 2.56), with a significant interaction (P for interaction < 0.001), indicating heightened sensitivity in hypertensive patients. DM: Diabetic patients (OR = 3.75) show a stronger RAR-CKM relationship, with higher risks (P for interaction < 0.001), suggesting diabetes exacerbates the link between RAR and CKM.

## Discussion

This study systematically evaluated the associations and predictive value of composite indices like RAR, NPAR, SIRI, and Homair with chronic kidney metabolic disease (CKM). Multimodal logistic regression (Table 3) showed that RAR consistently maintained a significant dose–response relationship across all adjusted models (model 3, OR: 2.73, 95% CI: 2.07–3.59, *p* < 0.001), with its effect size much higher than that of other indicators, suggesting that RAR may be a potential independent marker of CKM. This result may stem from the fact that RAR integrates the dual pathophysiological mechanisms of inflammation (24) and nutritional metabolism (25) (e.g., albumin), reflecting the synergistic effects of inflammation activation and metabolic imbalance in CKM. In model 3, the high exposure groups of NPARQ and SIRIQ (Q4) showed independent effects (OR: 1.41, 95% CI: 1.13–1.77, *p* < 0.001; OR: 1.47, 95% CI: 1.15–1.88, *p* < 0.001). However, the effects of the low exposure groups might be influenced by confounding factors. Restricted cubic spline (RCS) analysis further revealed the linear and nonlinear associations between RAR, NPAR, SIRI, and Homair in all three models with CKM, indicating that their predictive power appears to significantly increase beyond a specific threshold (P for overall: < 0.05), providing a theoretical basis for clinical risk stratification. In survival analysis, the high RAR group was associated with an increased risk for all-cause mortality, kidney disease mortality, and cardiovascular mortality. This supports the importance of RAR in prognostic evaluation (Log-rank *p* < 0.001).

Machine learning models [e.g., LightGBM (26)] achieved excellent performance (AUC = 0.92) with Lasso-selected variables (Supplementary Figure S3). SHAP analysis further identified RAR, age, and DM as core predictors (Figure 3). Dependence plots revealed concurrent elevations in RAR, age, and DM status. This suggests RAR’s role as a biomarker for the metabolic-inflammation vicious cycle, where elevated RAR may directly contributes to organ damage. In subgroup analysis (Supplementary Figure S7), significant interactions between RAR and education level, smoking, hypertension (HBP), DM, and age were observed (*p* < 0.05), indicating that its effect might be modulated by social behaviors and metabolic comorbidities, which might require differentiated consideration in interventions. The DCA curve also indicated that the model composed of these variables performed well, with net benefits significantly higher than those of the “no intervention” and “full intervention” scenarios at different CKM (event occurrence) threshold probabilities.

Previous studies have primarily focused on the association between single inflammation or metabolic biomarkers and CKM. For example, Yang et al. (27) investigated the relationship between the non-high-density lipoprotein cholesterol to high-density lipoprotein cholesterol ratio (NHHR) and CKM, finding that dyslipidemia and lipid metabolism abnormalities could be valuable in identifying high-risk individuals for CKM syndrome in its early stages. Similarly, Peng et al. (28) explored composite indicators like the triglyceride-glucose index (TyG), demonstrating that the combination of TG and FBG with other clinical data might help to predict the development and progression of CKM. Tang et al. (29), in their study of the Planetary Health Diet Index (PHDI), highlighted the close relationship between diet and CKM. Although many studies have investigated CKM prediction, there is limited exploration of integrated inflammation and nutritional-metabolic indices (e.g., RAR). This study, for the first time, suggests the central role of RAR in CKM risk stratification, which aligns with recent theories emphasizing the “inflammation-metabolism axis (30, 31)” in chronic diseases. Additionally, the relationship between other associated factors such as SIRI, NPAR, and Homair with CKM is explored, collectively investigating the predictive value of multiple biomarkers for CKM.

The innovative aspects of this study are as follows: First, it is the first to systematically compare the predictive performance of multiple inflammation-nutrition-metabolism composite indicators for CKM, identifying RAR as the most robust predictor; Second, it integrates traditional statistical methods (multivariable regression, RCS) with advanced machine learning techniques (LightGBM, SHAP) to validate findings from multiple perspectives, including associations, nonlinear effects, and predictive performance, thereby enhancing the reliability of the conclusions; and Third, it links biomarkers directly to clinical outcomes and decision-making benefits through survival analysis and DCA curves, facilitating their translation into clinical practice. With a large sample (*n* = 19,884) and rigorous confounder adjustment, this study minimizes potential bias and enhances result validity.

This study has several limitations. First, the reliance on retrospective self-reported data might introduce information bias and measurement errors, potentially leading to misclassification of CKM stages. Second, the inclusion of only PREVENT equation-related patients and the use of NHANES-derived CKM population limits the generalizability of our findings. Third, the cross-sectional design precludes causal inferences, necessitating validation in prospective cohorts to confirm the predictive value of RAR. Fourth, despite multiple imputations for missing data, unmeasured confounders (e.g., diet, medication use) might influence the results. Fifth, the machine learning models require external validation to confirm their applicability to diverse populations. Finally, some subgroup analyses (e.g., sex, race) did not reach statistical significance, possibly due to sample heterogeneity or insufficient statistical power, warranting further exploration. Future studies should address these limitations through prospective validation, external cohort testing, and more detailed subgroup analyses.

## Conclusion

This study demonstrates that RAR, as a composite inflammation-nutrition-metabolism indicator, is independently associated with CKM risk and prognosis, exhibiting significantly superior predictive performance compared to other biomarkers. By integrating machine learning models with the SHAP interpretability framework, RAR, age, and DM were identified as key predictors, providing a novel tool for early screening and risk stratification of CKM, further validated by decision curve analysis (DCA). Future research should focus on elucidating the biological mechanisms underlying RAR and exploring its clinical utility in dynamic monitoring, while also advancing the validation and optimization of multidimensional prediction models in real-world settings.

## Data Availability

The raw data supporting the conclusions of this article will be made available by the authors, without undue reservation.

## Glossary

CVD: Cardiovascular Disease
DM: Diabetes Mellitus
HBP: High Blood Pressure
CKD: Chronic Kidney Disease
FINS: Fasting Insulin
ALB: Albumin
UA: Uric Acid
CR: Creatinine
FBG: Fasting Blood Glucose
HbA1c: Hemoglobin A1c
WBC: White Blood Cell Count
NCP: Neutrophil Count Percentage
NC: Neutrophil Count
RDW: Red Cell Distribution Width
TC: Total Cholesterol
TG: Triglycerides
WC: Waist Circumference
Egfr: Estimated Glomerular Filtration Rate
LYC: Lymphocyte Count
MCC: Monocyte Count
PLT: Platelet Count
BMI: Body Mass Index
UACR: Urine Albumin-to-Creatinine Ratio
EDU: Education
PIR: Poverty Income Ratio
RAR: Red Cell Distribution Width to Albumin Ratio
NPAR: Neutrophil Percentage to Albumin Ratio
SIRI: Systemic Immune-Inflammation Index
Homair: Homeostatic Model Assessment for Insulin Resistance
eGFR: Estimated Glomerular Filtration Rate
RCS: restricted cubic splines
AUC: area under the ROC curve
