# Supplementary material for: Machine learning with decision curve analysis evaluates nutritional metabolic biomarkers for cardiovascular-kidney-metabolic risk: an NHANES analysis
Source: Front Nutr. 2025 May 8;12:1597864. doi: 10.3389/fnut.2025.1597864 (PMC12094989; doi:10.3389/fnut.2025.1597864)
Supplement: Supplementary file 1 [file Data_Sheet_1.docx]

Figure s1 Comparison of ROC curves between new and traditional biomarkers


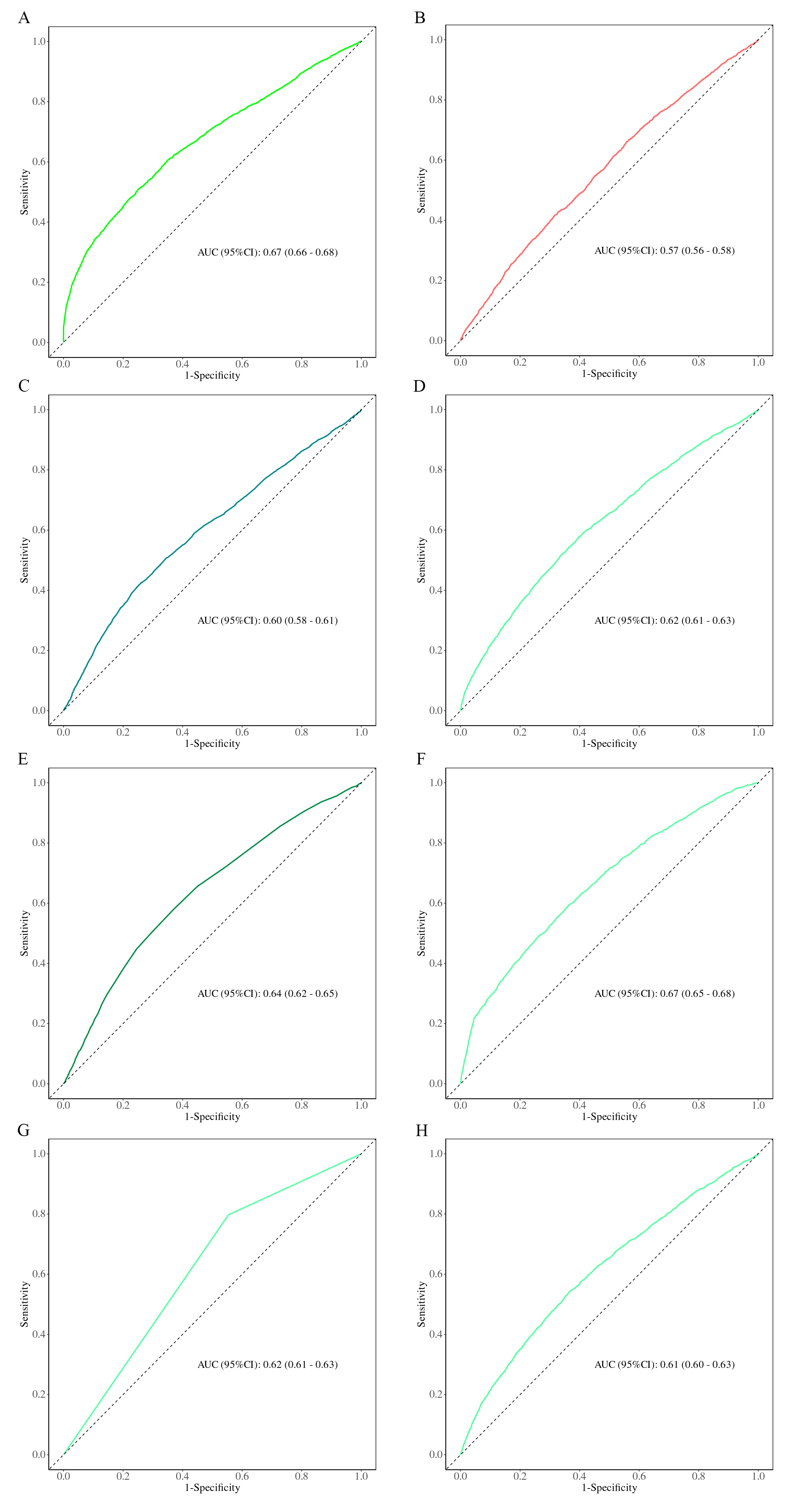


A: ROC of Egfr

B: ROC of Homair

C: ROC of FBG

D: ROC of NPAR

E: ROC of HbA1c

F: ROC of RAR

G: ROC of HBP

H: ROC of SIRI

Figure s2 The inclusion and exclusion process

101,316 participants from NHANES 2009-2018: Initial number of participants.

Exclude: Missing data for CKM definition (n=80,978): Excluded participants with missing data required for CKM definition.

Exclude: Age < 20 or Pregnant (n=454): Excluded participants under 20 years old or pregnant.

Final sample (N=19,884): Final number of participants included in the analysis.

Figure s3 Lasso Regression Results


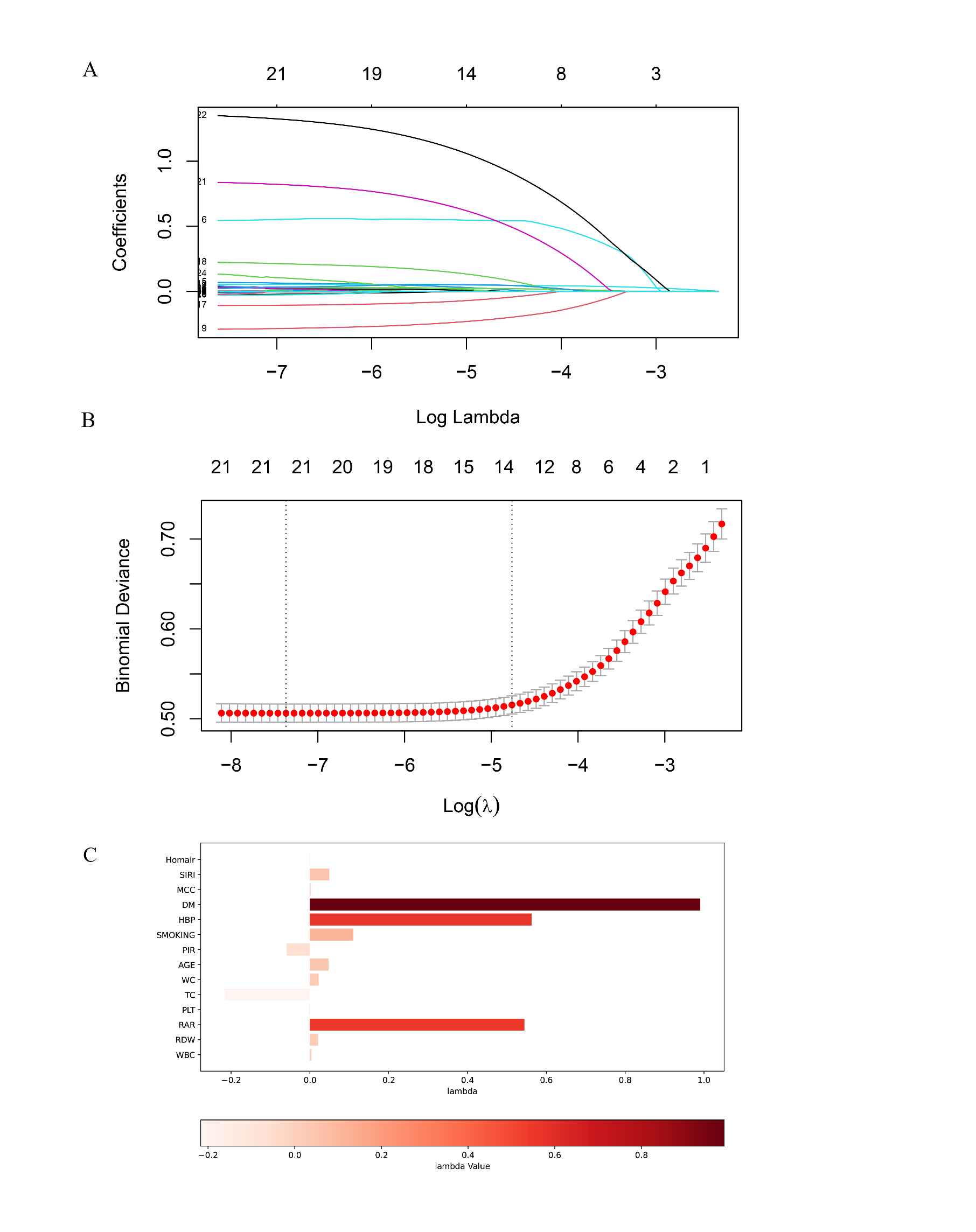


A: LASSO coefficient path as a function of log lambda. Each line represents the coefficient trajectory of a variable as the regularization parameter (lambda) changes

B: Binomial deviance plotted against log lambda. The red dots indicate model performance at each lambda value, and the vertical dotted line represents the selected lambda based on cross-validation

C: LASSO coefficients at the selected lambda value. The length of each bar corresponds to the magnitude of the variable's coefficient, with the color intensity representing the value of lambda. Variables with higher coefficients are more influential in the model

Figure s4 Comparison Between Validation Set and Test Set in Machine Learning Models


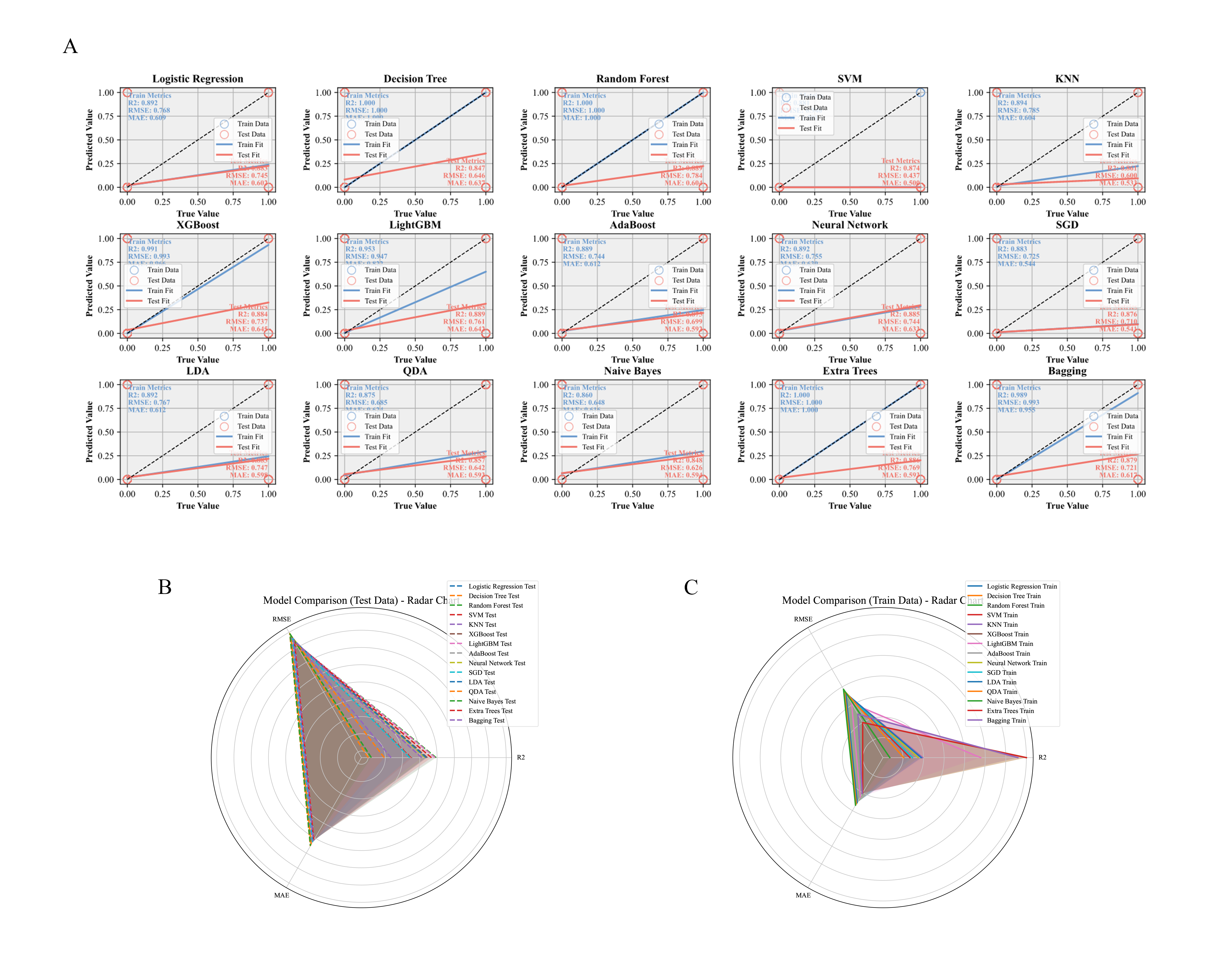


A: Predicted vs. True values for various machine learning models. The red circles represent data points from the training set, and the blue squares represent data points from the test set. The plots show how well the predicted values match the true values for both training and test data.

B: Radar chart comparing the performance of models on the test dataset. The performance metrics (R², RMSE, and MAE) are shown for each model, with lower RMSE and MAE indicating better performance.

C: Radar chart comparing the performance of models on the training dataset. The metrics are similar to Panel B, showing model performance during training. The results indicate that models such as XGBoost, LightGBM, and Neural Network exhibit strong generalization ability with low error rates on the test data, indicating no overfitting.

Figure s5 Machine Learning After Handling Missing Data


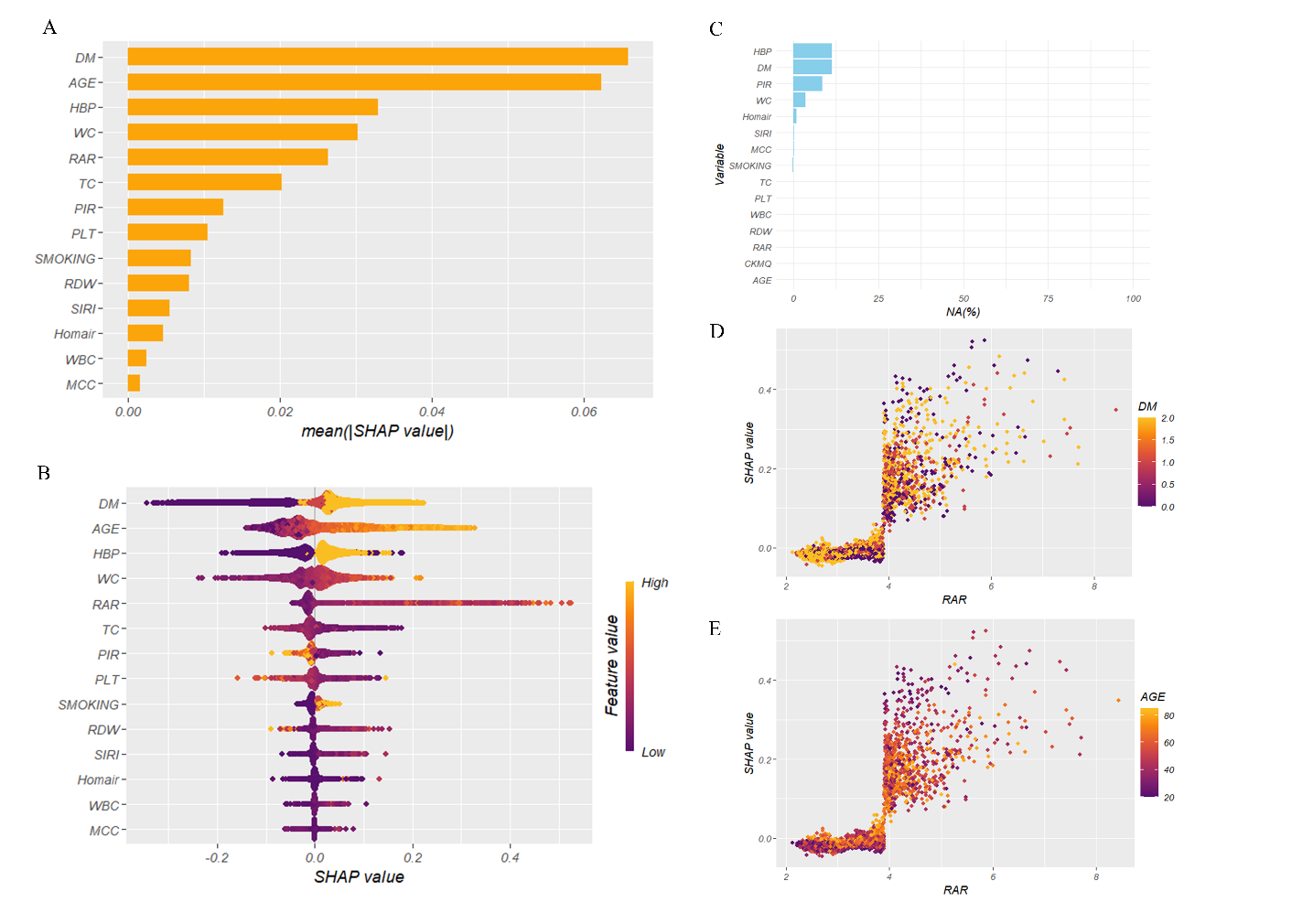


A: the feature importance plot

B: SHAP beeswarm plot

C: Missing date plot

D: the dependence plots for RAR-DM

E: the dependence plots for RAR-AGE

Figure s6 Calibration Curves
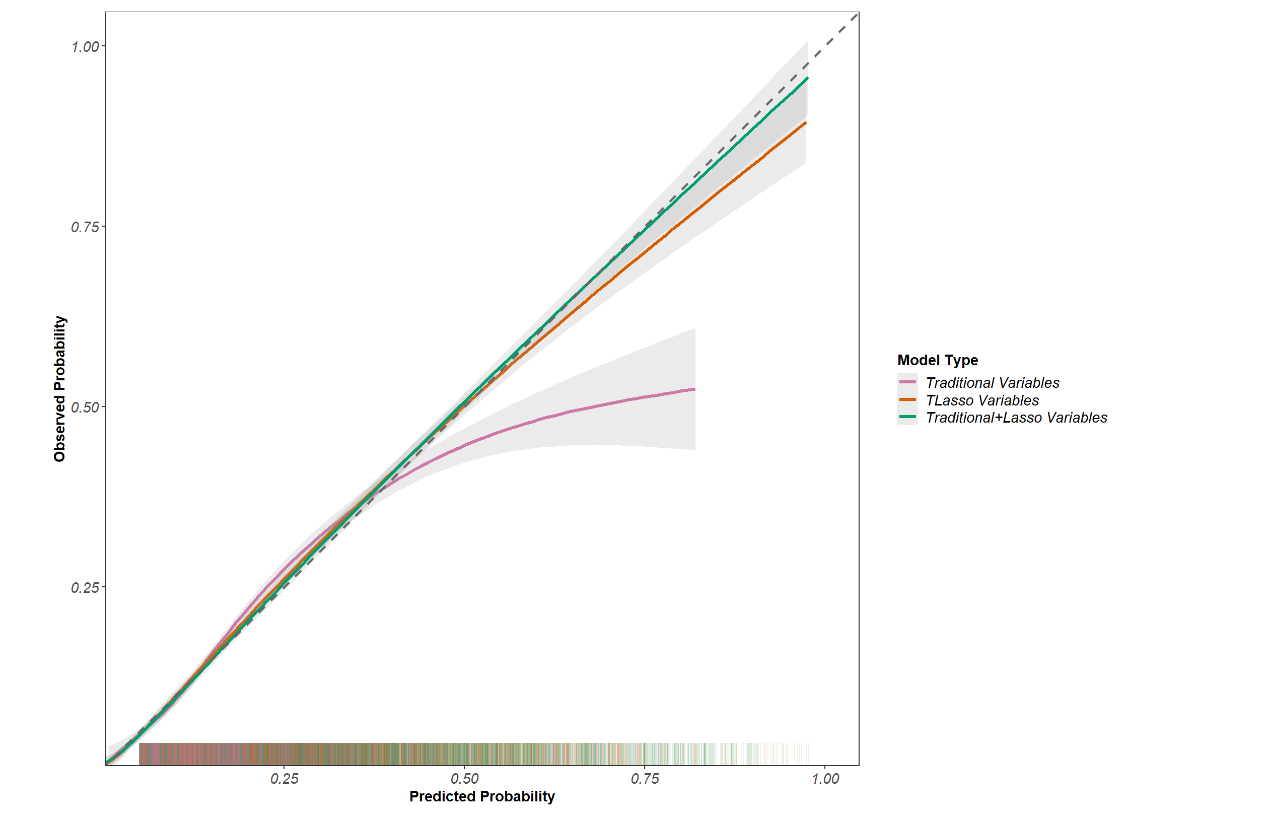


Calibration curve comparing the observed probability versus predicted probability for different models. The curves represent:

Traditional Variables (red): Model using only traditional variables.

Lasso Variables (orange): Model using only Lasso-selected variables.

Traditional + Lasso Variables (green): Model using both traditional and Lasso-selected variables.

The dashed line represents perfect calibration, where observed probabilities match predicted probabilities.The shaded areas indicate the confidence intervals for each model's performance.

Figure s7 Subgroup Analysis


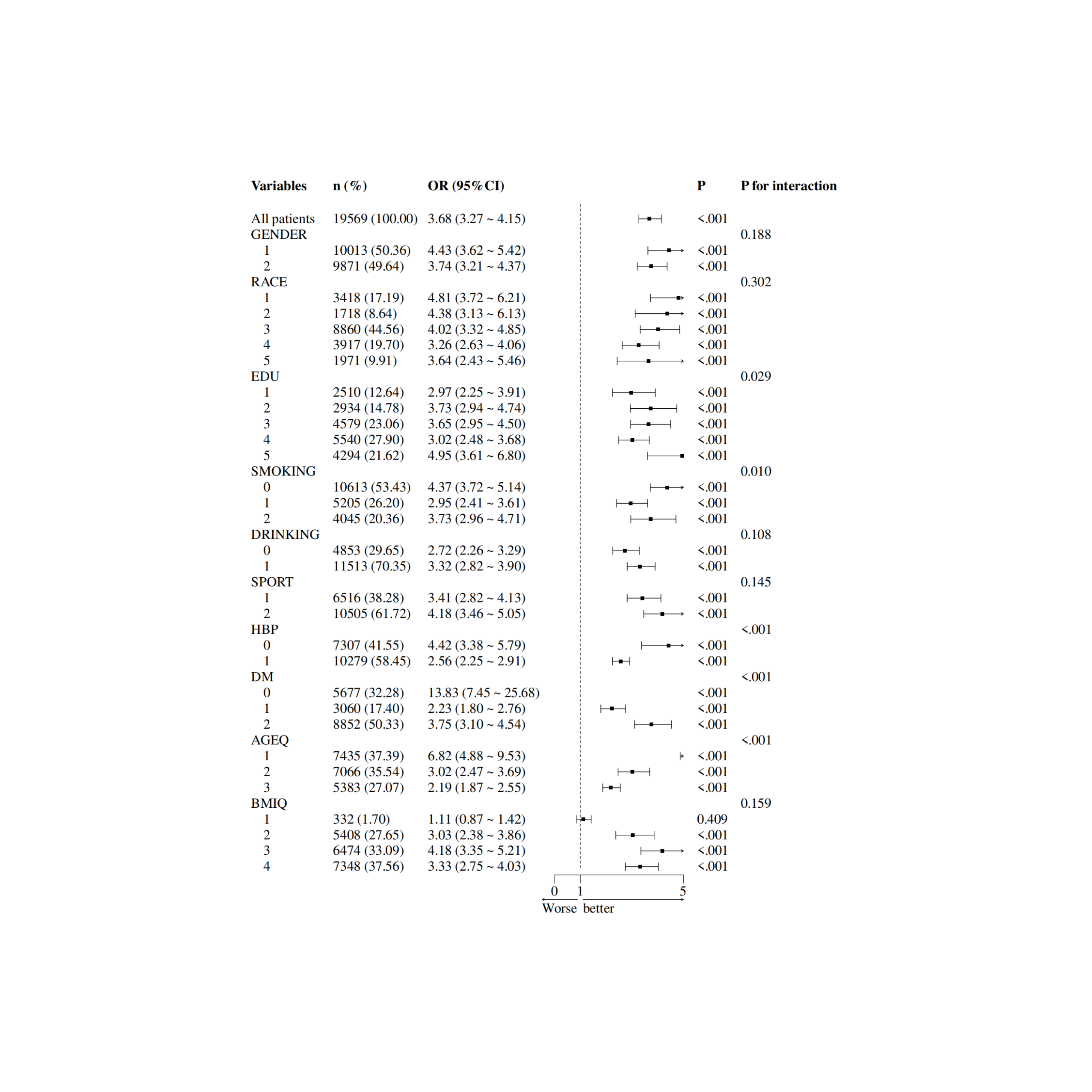


Forest plot showing the results of subgroup analysis for different variables, including odds ratios (OR) and their 95% confidence intervals (CI). Each horizontal line represents the OR and its 95% CI for the corresponding variable in each subgroup, with squares indicating the OR values and the horizontal lines representing the corresponding confidence intervals.

n (%): The sample size for each subgroup and its percentage in the total sample.

OR (95% CI): Odds ratios with 95% confidence intervals.

P: The p-value for the statistical significance of the variable within each subgroup.

P for interaction: The p-value for the interaction effect between variables, indicating the significance of the interaction term.

Dashed line: Represents the reference line where the OR equals 1. Values below this line indicate a smaller effect, while values above this line suggest a larger effect.
