# Supplementary material for: Machine learning with decision curve analysis evaluates nutritional metabolic biomarkers for cardiovascular-kidney-metabolic risk: an NHANES analysis
Source: Front Nutr. 2025 May 8;12:1597864. doi: 10.3389/fnut.2025.1597864 (PMC12094989; doi:10.3389/fnut.2025.1597864)
Supplement: Supplementary file 2 [file Data_Sheet_2.docx]

Table s1 Diagnostic Criteria and Categorical Variables for Health Conditions and Demographics

| **Condition** | **Diagnostic Criteria** |
| --- | --- |
| **CVD (Cardiovascular Disease)** | Participants who answered "Yes" to any of the following conditions: congestive heart failure, coronary heart disease, angina, myocardial infarction, COPD, or stroke. |
| **DM (Diabetes Mellitus)** | A history of diabetes, insulin use, oral diabetic medication for blood sugar reduction, HbA1c ≥ 6.5%, fasting blood glucose ≥ 126 mg/dL, or postprandial blood glucose ≥ 200 mg/dL. |
| **HBP (High Blood Pressure)** | A history of hypertension, SBP > 130 mmHg or DBP > 80 mmHg. |
| **CKD (Chronic Kidney Disease)** | eGFR < 60 mL/min/1.73 m² or urinary albumin-to-creatinine ratio (UACR) > 30 mg/g. |
| **AGE** | 1 (20-45 years), 2 (45-65 years), 3 (≥65 years) |
| **RACE** | 1) Mexican American, 2) Other Hispanic, 3) Non-Hispanic White, 4) Non-Hispanic Black, 5) Other |
| **MAYYR** | 1) Married, 2) Widowed, 3) Divorced, 4) Separated, 5) Never married |
| **EDUCATION** | 1) Less Than 9th Grade, 2) 9-11th Grade (Includes 12th grade with no diploma), 3) High School Grad/GED or Equivalent, 4) Some College or AA degree, 5) College Graduate or above |
| **SMOKING** | 0) No, 1) Yes |
| **DRINKING** | 0) No, 1) Yes |
| **BMI** | 1) ＜18(Underweight), 2)18-25 (Normal weight), 3) 25-30 (Overweight), 4) ≥30 (Obese) |
| **SPORT** | 1) <500 MET/wk, 2) ＞500 MET/wk |

**Table s2 CKM Syndrome Staging and Definitions**

| **Stage** | **Definition** |
| --- | --- |
| **Stage 0: No CKM Health Risk Factors** | Individuals without overweight/obesity, metabolic risk factors (hypertriglyceridemia, hypertension, diabetes, metabolic syndrome), chronic kidney disease (CKD), or subclinical/clinical cardiovascular disease (CVD).  **Must meet the following criteria:** (1) BMI between 18.5 and 25 kg/m² (inclusive) (2) Waist circumference (WC) <102 cm for males and <88 cm for females |
| **Stage 1: Excess or Dysfunctional Adipose Tissue** | Individuals with overweight/obesity, abdominal obesity, or dysfunctional adipose tissue but without other metabolic risk factors, CKD, or subclinical/clinical CVD.  **Must meet any of the following criteria:** (1) BMI ≥ 25 kg/m² (2) Waist circumference ≥ 102 cm for males and ≥ 88 cm for females (3) Fasting blood glucose (FBG) level between 100 and 124 mg/dL, or glycated hemoglobin (HbA1c) level between 5.7% and 6.4% |
| **Stage 2: Metabolic Risk Factors and Moderate-to-High-Risk CKD** | Individuals with metabolic risk factors (hypertriglyceridemia, hypertension, diabetes, metabolic syndrome) or moderate-to-high-risk stages of chronic kidney disease (CKD).  **Must meet any of the following criteria:** (1) Triglycerides (TG) > 135 mg/dL (2) Hypertension defined as systolic blood pressure (SBP) ≥130 mm Hg, diastolic blood pressure (DBP) ≥80 mm Hg, or a medical diagnosis, or use of antihypertensive medications (3) Diabetes defined as fasting blood glucose (FBG) >126 mg/dL, HbA1c ≥ 6.5%, a medical diagnosis, or use of insulin or hypoglycemic agents (4) Moderate-to-high-risk CKD according to KDIGO classification, defined as: - Urine albumin-to-creatinine ratio (UACR) ≥30 mg/g and eGFR ≥60 ml/min/1.73m², or - UACR <300 mg/g and eGFR between 45-59 ml/min/1.73m², or - UACR <30 mg/g and eGFR between 30-44 ml/min/1.73m² |
| **Stage 3: Subclinical Cardiovascular Disease (CVD) in CKM** | Subclinical risk equivalents for cardiovascular disease (CVD): High predicted 10-year CVD risk or very high-risk CKD stages according to KDIGO.  **Must meet any of the following criteria:** (1) High 10-year CVD risk defined as ≥20% risk, calculated using the basic PREVENT equation for CVD events (2) Very high-risk CKD according to KDIGO classification, defined as: - UACR ≥300 mg/g and eGFR ≤45-59 ml/min/1.73m², or - UACR ≥30 mg/g and eGFR ≤30-44 ml/min/1.73m², or - eGFR ≤29 ml/min/1.73m² |
| **Stage 4: Clinical Cardiovascular Disease (CVD) in CKM** | Individuals with diagnosed clinical cardiovascular disease (self-reported diagnoses, including heart failure, coronary artery disease, angina, heart attack, and stroke). |

**Notes:**

**CKM**: Cardiovascular-Kidney-Metabolic Syndrome.

**BMI**: Body Mass Index.

**WC**: Waist Circumference.

**FBG**: Fasting Blood Glucose.

**HbA1c**: Glycated Hemoglobin.

**TG**: Triglycerides.

**SBP**: Systolic Blood Pressure.

**DBP**: Diastolic Blood Pressure.

**CKD**: Chronic Kidney Disease.

**CVD**: Cardiovascular Disease.

**KDIGO**: Kidney Disease: Improving Global Outcomes.

**UACR**: Urine Albumin-to-Creatinine Ratio.

**eGFR**: Estimated Glomerular Filtration Rate.

Table s3 Comparison of Key Variables Across Non-DM, Pre-DM, and DM Groups of NHANES 1999–2018 participants, weighted for representativeness

| Variable | Total (n = 19884) | Non-DM (n=8883) | Pre-DM (n=8268) | DM (n=2709) | Statistic | *P* |
| --- | --- | --- | --- | --- | --- | --- |
|  |  |  |  |  |  |  |
| RAR, Mean (SE) | 3.11 (0.01) | 3.07 (0.01) | 3.11 (0.01) | 3.24 (0.01) | F=146.73 | **<.001** |
| NPAR, Mean (SE) | 13.74 (0.03) | 13.51 (0.04) | 13.78 (0.04) | 14.67 (0.07) | F=161.15 | **<.001** |
| SIRI, Mean (SE) | 1.21 (0.01) | 1.12 (0.01) | 1.27 (0.01) | 1.43 (0.03) | F=179.13 | **<.001** |
| Homair, Mean (SE) | 65.01 (0.86) | 39.03 (0.50) | 68.60 (0.92) | 170.16 (5.59) | F=839.83 | **<.001** |
| SE: Standard Error | | | | | | |
| F: ANOVA | | | | | | |

Pre-DM: HbA1c: 5.7%-6.4%, fasting blood glucose: 100-125 mg/dL.

DM: A history of diabetes, insulin use, oral diabetic medication for blood sugar reduction, HbA1c ≥ 6.5%, fasting blood glucose ≥ 126 mg/dL, or postprandial blood glucose ≥ 200 mg/dL.

Abbreviations: RAR (Red Cell Distribution Width to Albumin Ratio), NPAR (Neutrophil Percentage to Albumin Ratio), SIRI (Systemic Immune-Inflammation Index), Homair (Homeostatic Model Assessment for Insulin Resistance)

Table s4 Comparison of Key Variables Between Non-CVD and CVD Groups of NHANES 1999–2018 participants, weighted for representativeness

| Variable | Total (n = 19884) | Non-CVD (n=15505) | CVD (n=2087) | Statistic | *P* |
| --- | --- | --- | --- | --- | --- |
|  |  |  |  |  |  |
| RAR, Mean (SE) | 3.11 (0.01) | 3.03 (0.01) | 3.35 (0.02) | t=15.03 | **<.001** |
| NPAR, Mean (SE) | 13.74 (0.03) | 13.55 (0.03) | 14.73 (0.09) | t=11.46 | **<.001** |
| SIRI, Mean (SE) | 1.21 (0.01) | 1.18 (0.01) | 1.56 (0.03) | t=11.94 | **<.001** |
| Homair, Mean (SE) | 65.01 (0.86) | 61.99 (0.94) | 85.46 (3.56) | t=6.20 | **<.001** |
| SE: Standard Error | | | | | |
| t: t-test | | | | | |

Abbreviations: RAR (Red Cell Distribution Width to Albumin Ratio), NPAR (Neutrophil Percentage to Albumin Ratio), SIRI (Systemic Immune-Inflammation Index), Homair (Homeostatic Model Assessment for Insulin Resistance)

Table s5 Summary of NHANES 1999–2018 Variables (P25, P50, P75) Stratified by Group Names

| Variable | P25 | P50 | P75 | Group Variable Name |
| --- | --- | --- | --- | --- |
| Homair | 26.1 | 43.44 | 74.389 | HomairQ |
| SIRI | 0.69 | 1 | 1.467 | SIRIQ |
| NPAR | 12.045 | 13.659 | 15.311 | NPARQ |
| RAR | 2.804 | 3.023 | 3.293 | RARQ |

Abbreviations: RAR (Red Cell Distribution Width to Albumin Ratio), NPAR (Neutrophil Percentage to Albumin Ratio), SIRI (Systemic Immune-Inflammation Index), Homair (Homeostatic Model Assessment for Insulin Resistance)

Table s6 Baseline characteristics stratified by RAR quartiles of NHANES 1999–2018, weighted for representativeness

| Variable | Total (n = 19884) | 1 (n=4036) | 2 (n=4593) | 3 (n=5013) | 4 (n=5927) | Statistic | *P* |
| --- | --- | --- | --- | --- | --- | --- | --- |
|  |  |  |  |  |  |  |  |
| CKMQ, n(%) |  |  |  |  |  | χ²=1021.77 | **<.001** |
| 0 | 1881 (11.33) | 579 (16.37) | 453 (11.67) | 421 (10.16) | 394 (7.32) |  |  |
| 1 | 2666 (14.40) | 529 (12.43) | 631 (15.13) | 642 (14.30) | 827 (15.70) |  |  |
| 2 | 11712 (58.03) | 2606 (63.79) | 2868 (60.38) | 3013 (57.91) | 3050 (50.52) |  |  |
| 3 | 1298 (6.94) | 151 (4.01) | 267 (6.40) | 370 (8.41) | 485 (8.56) |  |  |
| 4 | 2327 (9.30) | 171 (3.41) | 374 (6.42) | 567 (9.23) | 1171 (17.91) |  |  |
| GENDER, n(%) |  |  |  |  |  | χ²=1034.48 | **<.001** |
| 1 | 10013 (49.93) | 2669 (65.45) | 2611 (54.97) | 2376 (46.14) | 2239 (34.50) |  |  |
| 2 | 9871 (50.07) | 1367 (34.55) | 1982 (45.03) | 2637 (53.86) | 3688 (65.50) |  |  |
| RACE, n(%) |  |  |  |  |  | χ²=792.36 | **<.001** |
| 1 | 3418 (8.01) | 823 (8.19) | 884 (8.27) | 855 (7.93) | 823 (7.90) |  |  |
| 2 | 1718 (5.48) | 274 (4.57) | 400 (5.41) | 493 (5.81) | 523 (6.05) |  |  |
| 3 | 8860 (68.15) | 2107 (74.55) | 2195 (71.23) | 2282 (69.13) | 2169 (58.48) |  |  |
| 4 | 3917 (11.12) | 347 (4.57) | 642 (7.73) | 928 (10.76) | 1890 (20.66) |  |  |
| 5 | 1971 (7.24) | 485 (8.12) | 472 (7.36) | 455 (6.37) | 522 (6.91) |  |  |
| EDU, n(%) |  |  |  |  |  | χ²=188.15 | **<.001** |
| 1 | 2510 (6.47) | 428 (5.02) | 603 (6.23) | 642 (6.61) | 777 (7.75) |  |  |
| 2 | 2934 (11.55) | 552 (10.11) | 611 (10.96) | 743 (11.64) | 977 (13.51) |  |  |
| 3 | 4579 (24.32) | 918 (23.59) | 1030 (23.47) | 1154 (24.24) | 1400 (25.78) |  |  |
| 4 | 5540 (30.50) | 1093 (30.05) | 1232 (29.09) | 1412 (31.42) | 1737 (31.80) |  |  |
| 5 | 4294 (27.16) | 1040 (31.24) | 1113 (30.24) | 1057 (26.09) | 1023 (21.15) |  |  |
| SMOKING, n(%) |  |  |  |  |  | χ²=13.31 | 0.248 |
| 0 | 10613 (52.86) | 2191 (53.78) | 2445 (53.30) | 2658 (51.83) | 3145 (52.26) |  |  |
| 1 | 5205 (26.12) | 1001 (25.05) | 1274 (26.97) | 1334 (26.81) | 1516 (25.78) |  |  |
| 2 | 4045 (21.02) | 841 (21.16) | 870 (19.72) | 1015 (21.36) | 1258 (21.96) |  |  |
| DRINKING, n(%) |  |  |  |  |  | χ²=406.29 | **<.001** |
| 0 | 4853 (25.18) | 781 (18.20) | 1075 (22.39) | 1315 (26.86) | 1587 (35.46) |  |  |
| 1 | 11513 (74.82) | 2927 (81.80) | 2999 (77.61) | 2865 (73.14) | 2573 (64.54) |  |  |
| SPORT, n(%) |  |  |  |  |  | χ²=333.35 | **<.001** |
| 1 | 6516 (34.32) | 959 (26.40) | 1343 (31.69) | 1645 (34.69) | 2446 (43.29) |  |  |
| 2 | 10505 (65.68) | 2423 (73.60) | 2554 (68.31) | 2653 (65.31) | 2749 (56.71) |  |  |
| CKD, n(%) |  |  |  |  |  | χ²=524.11 | **<.001** |
| 0 | 15228 (93.27) | 3687 (97.47) | 3929 (95.62) | 3880 (91.83) | 3679 (86.50) |  |  |
| 1 | 1493 (6.73) | 124 (2.53) | 234 (4.38) | 431 (8.17) | 701 (13.50) |  |  |
| CRS, n(%) |  |  |  |  |  | χ²=343.34 | **<.001** |
| 0 | 16173 (97.64) | 3771 (99.32) | 4099 (98.91) | 4169 (97.49) | 4078 (94.02) |  |  |
| 1 | 547 (2.36) | 40 (0.68) | 64 (1.09) | 141 (2.51) | 302 (5.98) |  |  |
| CVD, n(%) |  |  |  |  |  | χ²=744.61 | **<.001** |
| 0 | 15505 (90.51) | 3808 (96.52) | 3985 (93.14) | 3910 (89.39) | 3574 (80.78) |  |  |
| 1 | 2087 (9.49) | 171 (3.48) | 374 (6.86) | 567 (10.61) | 931 (19.22) |  |  |
| HBP, n(%) |  |  |  |  |  | χ²=348.76 | **<.001** |
| 0 | 7307 (46.02) | 2058 (53.58) | 1990 (49.68) | 1736 (43.07) | 1451 (35.77) |  |  |
| 1 | 10279 (53.98) | 1918 (46.42) | 2369 (50.32) | 2742 (56.93) | 3050 (64.23) |  |  |
| DM, n(%) |  |  |  |  |  | χ²=856.28 | **<.001** |
| 0 | 5677 (33.66) | 1049 (27.28) | 1413 (34.26) | 1566 (37.45) | 1569 (36.97) |  |  |
| 1 | 3060 (13.35) | 386 (7.19) | 585 (10.80) | 790 (14.30) | 1202 (22.38) |  |  |
| 2 | 8852 (52.99) | 2544 (65.52) | 2361 (54.94) | 2122 (48.25) | 1730 (40.65) |  |  |
| AGEQ, n(%) |  |  |  |  |  | χ²=1122.50 | **<.001** |
| 1 | 7435 (43.26) | 2264 (59.95) | 1841 (44.59) | 1568 (36.49) | 1678 (32.78) |  |  |
| 2 | 7066 (37.09) | 1207 (31.13) | 1654 (38.07) | 1934 (40.94) | 2153 (38.19) |  |  |
| 3 | 5383 (19.66) | 565 (8.93) | 1098 (17.33) | 1511 (22.57) | 2096 (29.04) |  |  |
| BMIQ, n(%) |  |  |  |  |  | χ²=1431.23 | **<.001** |
| 1 | 332 (1.86) | 121 (3.16) | 81 (1.78) | 61 (1.37) | 59 (0.97) |  |  |
| 2 | 5408 (28.98) | 1612 (41.51) | 1356 (30.15) | 1248 (25.42) | 1106 (19.11) |  |  |
| 3 | 6474 (32.38) | 1457 (35.09) | 1675 (35.93) | 1690 (32.39) | 1569 (26.28) |  |  |
| 4 | 7348 (36.78) | 816 (20.23) | 1434 (32.13) | 1945 (40.82) | 3026 (53.64) |  |  |
| χ²: Chi-square test | | | | | | | |
| BMI (Body Mass Index) | | | | | | | |

CVD (Cardiovascular Disease)

DM (Diabetes Mellitus)

HBP (High Blood Pressure)

CKD (Chronic Kidney Disease)

Table s7 Binary Logistic Regression Analysis with Optimal Cutoff Values for Key Variables

| Variable | Node 1  OR (95% CI) | Node 1  P-value | Node 2  OR (95% CI) | Node 2  P-value | Cutoff Value |
| --- | --- | --- | --- | --- | --- |
| RAR | 0.33 (0.28 ~ 0.37) | <.001 | 3.07 (2.67 ~ 3.54) | <.001 | 2.977 |
| NPAR | 1.84 (0.40 ~ 8.50) | <.001 | 2.04 (1.80 ~ 2.30) | <.001 | 13.533 |
| SIRI | 0.52 (0.46 ~ 0.59) | 0.438 | 1.92 (1.69 ~ 2.18) | <.001 | 1.000 |
| Homair | 0.57 (0.50 ~ 0.64) | <.001 | 1.77 (1.56 ~ 2.00) | <.001 | 43.008 |

Abbreviations: RAR (Red Cell Distribution Width to Albumin Ratio), NPAR (Neutrophil Percentage to Albumin Ratio), SIRI (Systemic Immune-Inflammation Index), Homair (Homeostatic Model Assessment for Insulin Resistance)
